# All-photonic switching of a benzo[*e*]-fused dimethyldihydropyrene–azobenzene dyad in the solid-state for logic operations†

**DOI:** 10.1039/d5sc02467f

**Published:** 2025-06-18

**Authors:** Sariful Molla, Jakir Ahmed, Subhajit Bandyopadhyay

**Affiliations:** a Department of Chemical Sciences Indian, Institute of Science Education and Research Kolkata Nadia 741246 Mohanpur WB India sb1@iiserkol.ac.in

## Abstract

Molecular photoswitches are crucial components for developing high-density memory devices and photonic information processing systems. A molecular photoswitch can generate at least two distinct states. Photochromic dyads of coupled photoswitches can attain more than two states if the two switches are independently addressable. However, orthogonal photoswitching in hybrid photochromic systems could be challenging. In this work, we discuss the development of two orthogonal hybrid photochromic dyads integrating two distinct photoswitches: dimethyldihydropyrene (DHP) or benzo[*e*]-fused dimethyldihydropyrene (BDHP) with azobenzene. Despite the significant spectral overlap between these systems, careful design and selection of suitable light sources ranging from NIR to UV light have successfully decoupled the individual photoswitching processes. As a result, four distinct well-characterized states can be selectively controlled with light. We constructed an all-photonic molecular logic gate by switching the system in a thin film, showcasing the potential of these systems for advanced molecular information processing using attenuated total reflectance (ATR)-based FTIR spectroscopy as a non-destructive readout mode.

## Abstract 2

Precise control of multi-state switching has been achieved through interplay of light in a benzo[*e*]-fused dimethyldihydropyrene–azobenzene photochromic dyad.

## Graphical abstract

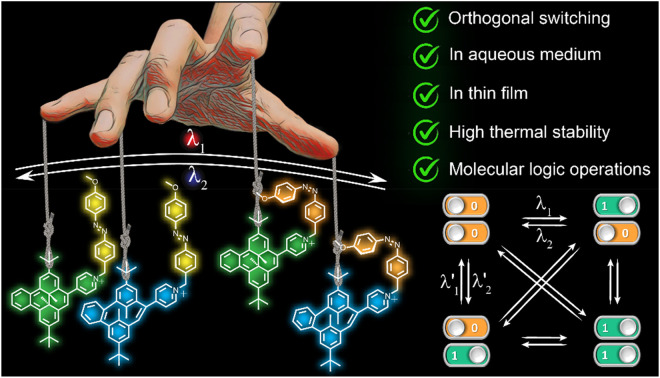

## Introduction

**Fig. 1:**
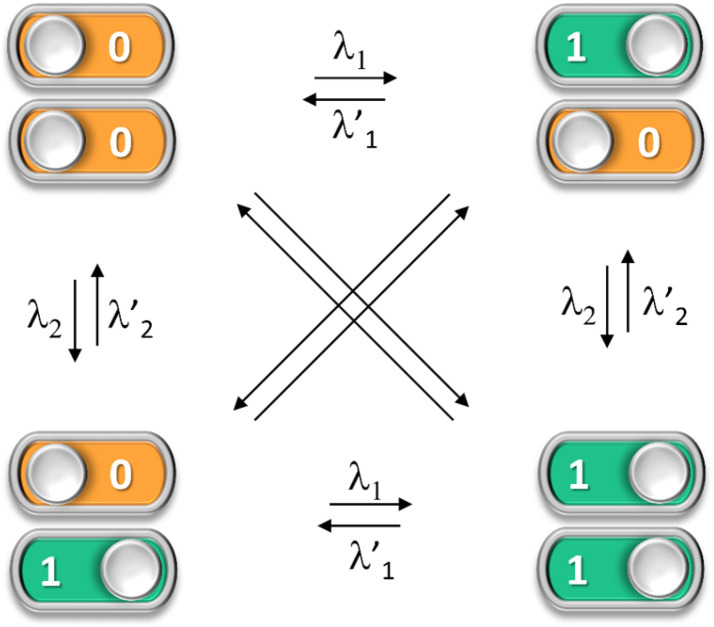
Multistate photoswitching in an ideal photochromic dyad. Isomerization between the two diagonal states would require a combination of two different wavelengths of light.

**Fig. 2:**
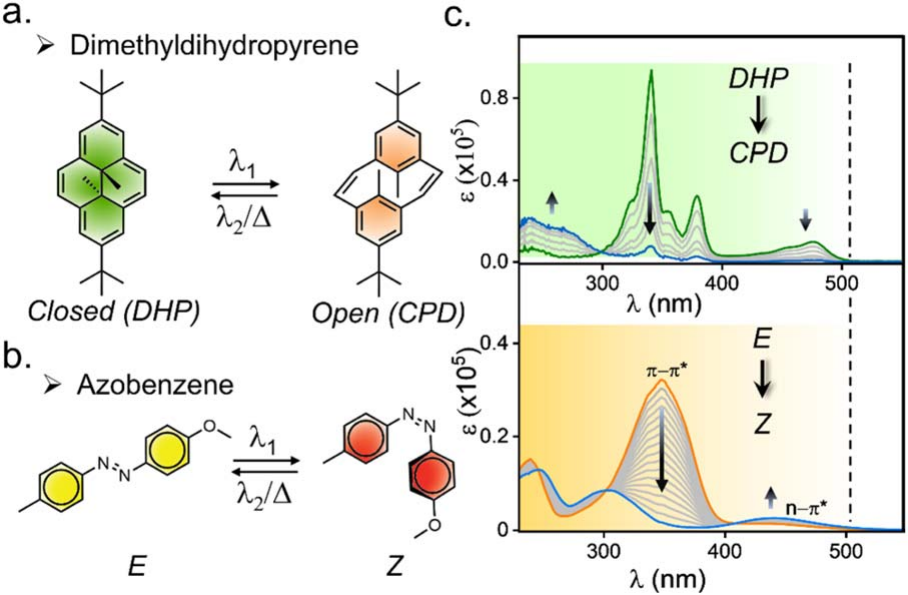
(a) Molecular structure and switching of dimethyldihydropyrene (DHP) and (b) azobenzene; (c) absorbance spectra of DHP and azobenzene, and the spectra upon isomerization, showcasing overlapping of the spectrum ranging from 230 nm to 510 nm in CH_3_CN.

Given the precise control of ON/OFF activity in different environments, ranging from the macroscale to the nanoscale, molecular switches have attracted significant attention in recent years.^1–6^ Particularly, the use of light as an external stimulus is of key importance because of its non-invasive nature and its ability to control processes both in terms of precise 3D space and time. Moreover, light does not generate any byproducts, unlike a chemical stimulus such as protons.^7^ The control of various processes in chemical research ranging from catalysis^8–11^ to photopharmacology,^12–15^ super-resolution fluorescence imaging,^16–19^ energy storage materials^20–24^ and the construction of molecular logic gates^25–28^ can be controlled reversibly by photochromic switches, such as stilbene, azobenzene, diarylethene, or dimethyldihydropyrene. A major advancement in the field with the emergence of several new generations of photoswitches including modified indigos,^29–32^ all-visible-light-enabled azobenzenes,^33–36^ heteroaryl azoswitches,^37–40^ donor–acceptor adducts (DASA),^41–43^ imidazole dimers^44–46^ and photoswitchable imines^47–49^ has provided a new impetus to the field. These monomeric photoswitches offer an ON/OFF binary response depending on the wavelength of the light used for the irradiation. However, the complexity of advanced devices demands the development of multi-responsive systems with more than two addressable states. In this regard, the emergence of multi-photochromic systems has offered novel solutions to the problem.^25,50–54^ Ideally, a multi-photochromic compound consisting of two photoswitches (a dyad) generates 2^2^ molecular states. Thus, a molecule having *n* photoswitches can generate 2^*n*^ states. To elicit the functions of the photoswitchable components in a multi-switch, each of the photoswitchable units of the molecule must be driven by orthogonal light sources that offer selective addressability of the individual states. To establish an all-photon photochromic system consisting of two components, at least four different lights are needed for the molecule to provide controllable access and selectively yield all four distinct states (Fig. 1). To make use of those states, they should be long-lived and stable under ambient conditions. Additionally, the efficiency of each photoconversion is determined by the composition of the photostationary states (PSS); a quantitative conversion, earmarked by a high PSS, results in more exclusive features and greater purity of a specific state. Achieving wavelength-orthogonality in a photoswitchable dyad requires distinct spectral separation for each constituent switch. However, the available molecular photoswitches, such as stilbene, azobenzene, dimethyldihydropyrene, diarylethenes, and spiropyrans have significant spectral overlap in the ultraviolet region,^3,55–58^ thus making it difficult to design a multi-photoswitch system consisting of these molecular switches. Recently, notable advancements have been made to achieve path-independent orthogonal photoswitching, but many of them require incorporation of a secondary stimulus such as a thermal,^50,59–61^ electrical, or chemical^62,63^ isomerization process to achieve orthogonality. In this work, we established all-photon, wavelength-selective orthogonal photoswitching by combining two classes of photochromic systems. Dimethyldihydropyrene (DHP) and azobenzene overlap in the UV-visible spectrum and use nearly identical wavelengths of light for photochemical isomerization (Fig. 2a–c). This makes it almost impossible to develop a hybrid photochromic dyad consisting of these two photoswitches that can be controlled orthogonally. To overcome this challenge, we introduced synthetic modifications on the parent DHP, resulting in a red-shifted UV-visible spectrum that enabled selective excitation. DHP, upon isomerization, can also react with molecular oxygen, reducing the photochromic efficiency.^64,65^ Again, synthetic measures have been taken to improve the switching efficiency in both water and thin films. The orthogonally photo-addressable states enabled the demonstration of a molecular logic device. Molecular logic gates are the prototypes for developing molecular information storage or molecular computing devices. Hybrid systems satisfy the fundamental requirements to establish a molecular logic gate, namely, (a) the output or the responses are easily observable, (b) the response is rapid, and (c) they exhibit reversibility or the ability to run multiple cycles without obvious fatigue.^66,67^ In this context, several readout-method-based molecular logic gates have been reported; for example, absorbance-probe-based,^68^ fluorescence-output-based,^69^ and more recently, SERS-output-based^70^ molecular logic gates have been developed. All these methods have their advantages and disadvantages. However, ATR-FTIR spectroscopy has never been used for molecular logic representations. The great advantage of ATR measurement is that it does not require extensive sample preparation; a high-quality IR spectrum can be obtained from a simple drop-casted sample. To the best of our knowledge, the present report is the first example of ATR FT-IR spectrum-based molecular logic operations.

## Results and discussion

**Fig. 3:**
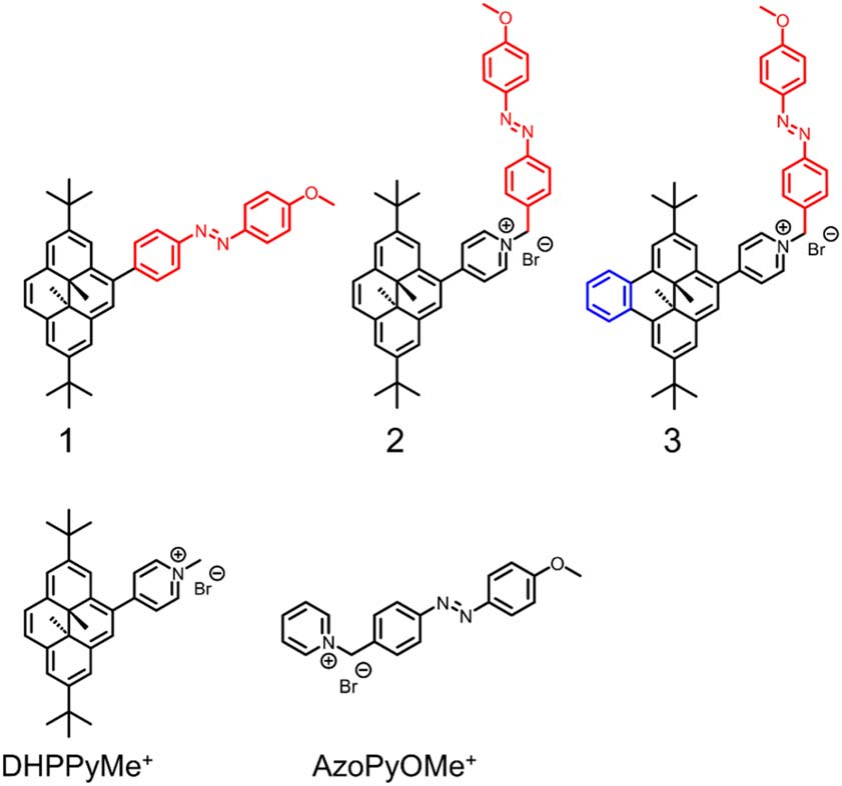
Studied derivatives.

**Fig. 4:**
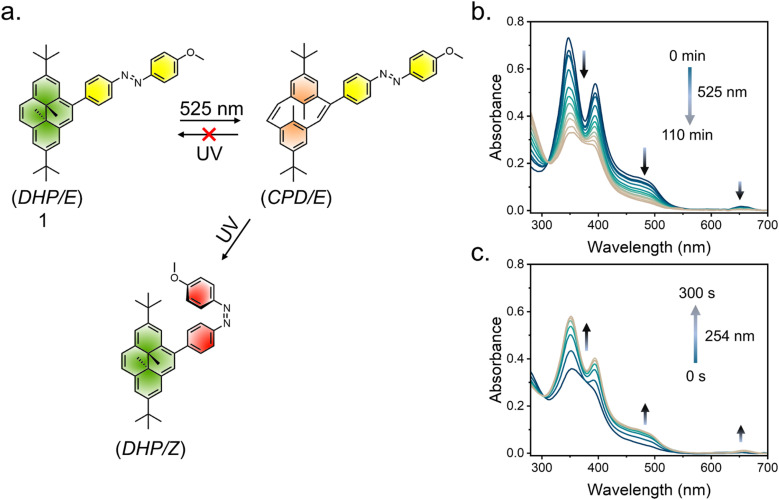
(a) Schematic of photoisomerization in derivative 1 upon irradiation with different lights. Changes in the UV/vis absorbance spectra with the (b) selective closed-to-open (DHP → CPD) isomerization of the DHP unit and (c) both the ring-closing of DHP (CPD → DHP) and the *E* → *Z* isomerization of the azobenzene component upon UV light irradiation.

**Fig. 5:**
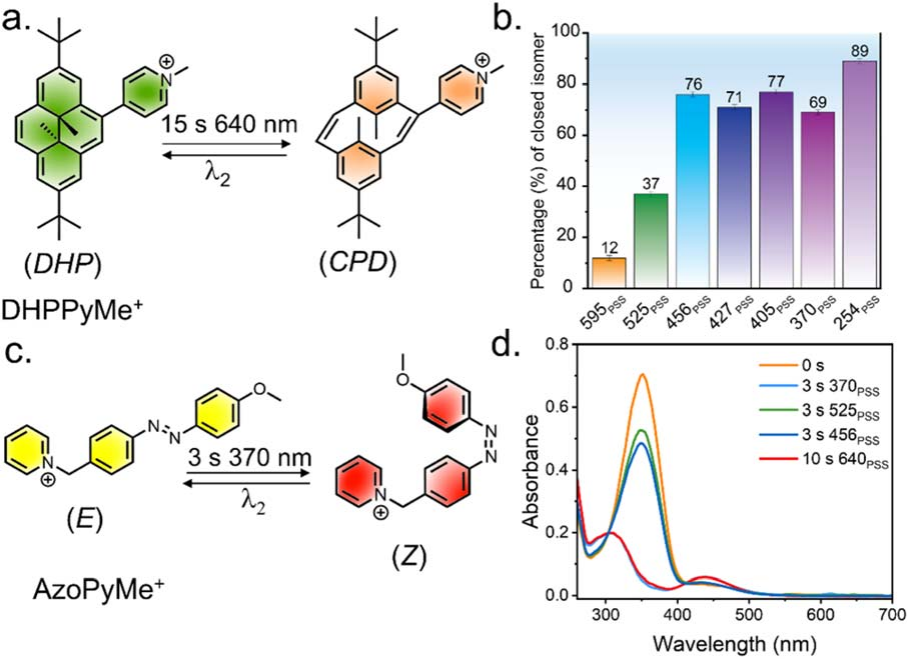
(a) Molecular structure of DHP with attached pyridinium acceptor. (b) Percentage of ring-closed isomer obtained during reverse isomerization for DHPPyMe^+^, showing the PSS upon illumination with lights of different wavelengths. (c) Molecular structure of azobenzene derivative for control studies. (d) UV/vis absorbance spectra of AzoPyMe^+^ upon irradiation with different lights. All the above-mentioned solution-state studies were carried out in acetonitrile solution (10 μM, 298 K).

**Fig. 6:**
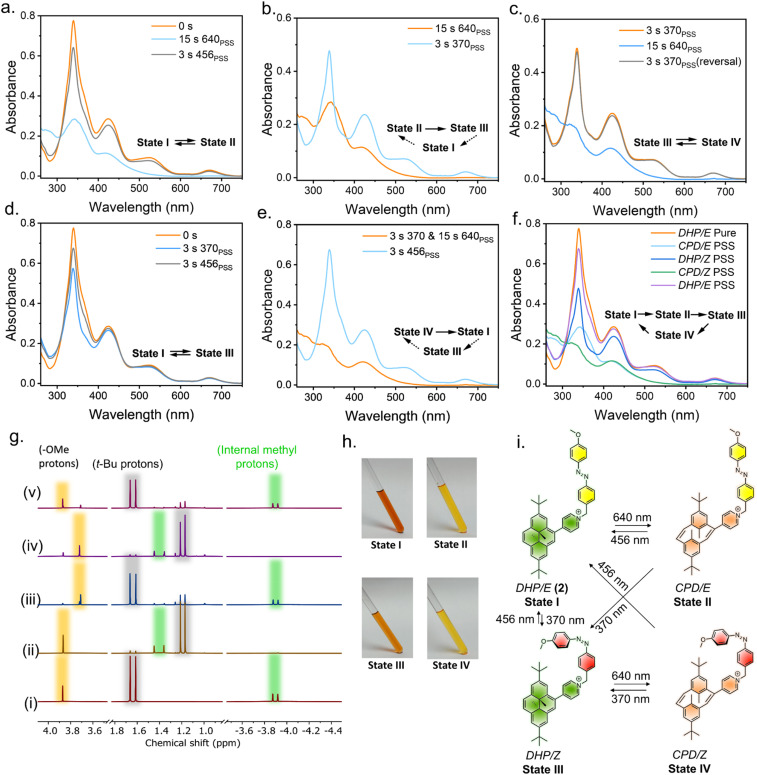
(a) Reversible and orthogonal isomerization of DHP (DHP/CPD) without affecting the azobenzene switch by irradiation with 640 nm light for 15 s and 456 nm light for 3 s. (b) Simultaneous switching of DHP (CPD → DHP) and azobenzene (*E* → *Z*) by 370 nm irradiation for 3 s. (c) Reversible control of the DHP/CPD isomerization keeping the *Z*-isomer of azobenzene intact by irradiation with 640 nm light for 15 s and 370 nm light for 3 s. (d) Reversible and orthogonal control of azobenzene switching (*E*/*Z*) without affecting the DHP switch by alternate irradiation of 370 nm (*E* → *Z*) and 456 nm (*Z* → *E*) light for 3 s. (e) Simultaneous reversal of CPD/*Z* → DHP/*E* by 456 nm irradiation for 3 s. (f) Sequential control of the different states and the corresponding spectra of PSS achieved by irradiation with different wavelengths of light. (g) Orthogonal photoswitching monitored *via*^1^H NMR (400 MHz, CD_3_CN, 298 K) studies with a 50 mM solution: (i) before irradiation (State I), and (ii) PSS of State II, depicting the shift of the internal methyl protons (green) and *t*-Bu protons (grey). (iii) PSS of State III highlighting the *E* → *Z* isomerization of the azobenzene with shift of the methoxy proton (yellow). (iv) PSS of State IV, and (v) recovery of State I with a PSS. (h) Photographs of 2 in different states in a 50 mM solution (CD_3_CN). Changes in the UV/vis spectrum upon photoexcitation of light at selective wavelengths. (i) Schematic representing all the possible all-photon orthogonal photoswitching achieved using a combination DHP–azobenzene coupled system with high PSS.

**Fig. 7:**
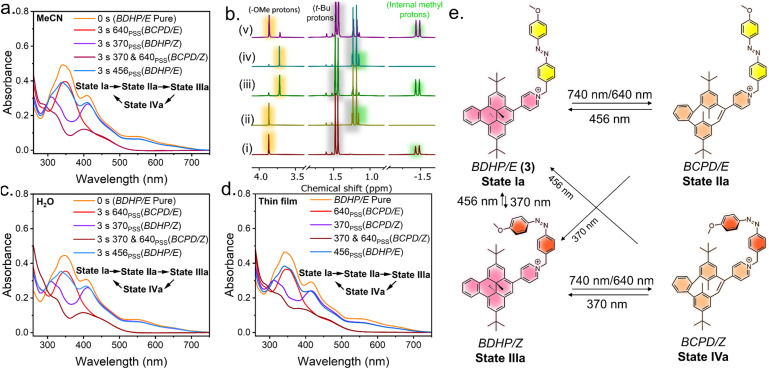
(a) Sequential control of different states and the corresponding absorbance spectra of the PSS achieved by irradiation with different wavelengths of light in MeCN (10 μM). (b) Orthogonal photoswitching monitored *via*^1^H NMR (400 MHz, CD_3_CN, 298 K) studies for a 50 mM solution: (i) before irradiation (State Ia). (ii) PSS of State IIa depicting the shift of the internal methyl protons (green) and *t*-Bu protons (grey). (iii) PSS of State IIIa highlighting the *E* → *Z* isomerization of the azobenzene with the shift of the methoxy protons (yellow). (iv) PSS of State IVa, and (v) recovery of State Ia with a PSS. Sequential control of the different states and the corresponding spectra of PSS achieved by irradiation with different wavelengths of light (c) in H_2_O (10 μM) and (d) in drop casted-thin films. (e) Schematic representing all the possible all-photon orthogonal photoswitching routes achieved using the combination BDHP–azobenzene coupled system with high PSS.

**Fig. 8:**
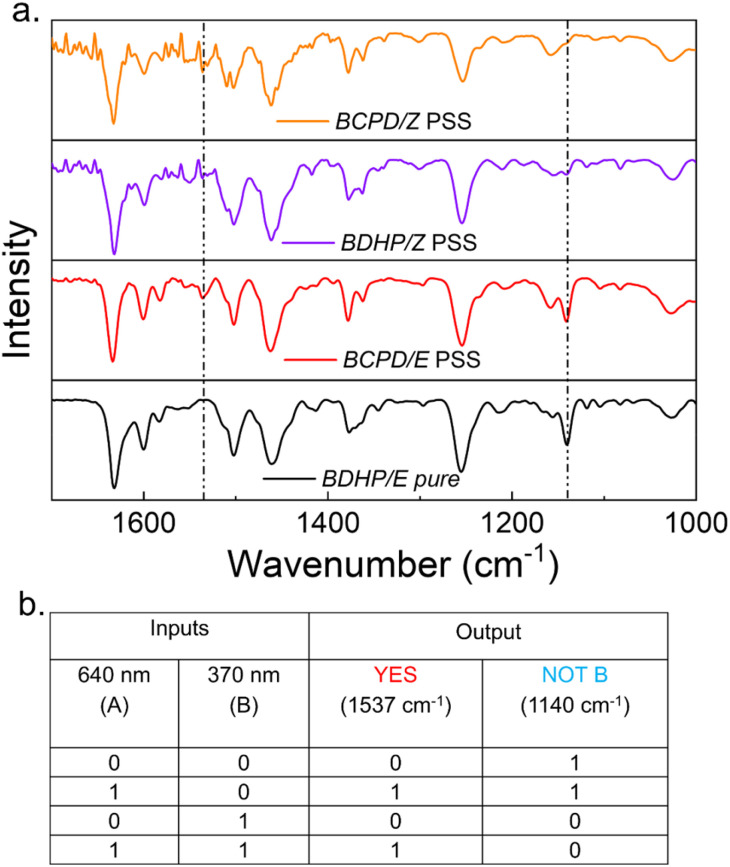
(a) Experimental ATR FTIR spectra of derivative 3 in the pristine state and other photogenerated states. (b) I/O truth table representing the optical input and the FTIR signal as the output.

The orthogonal photochromic systems discussed here consist of dimethyldihydropyrene (DHP), a negative photochromic system, and azobenzene, a positive photochromic system, each utilizing distinct photoisomerization mechanisms. Our initial investigations focused on a relatively simple system 1, in which DHP and azobenzene are electronically conjugated (Fig. 3). A Suzuki–Miyaura coupling reaction gave the final derivative (see ESI† for synthetic details). The two UV/vis-responsive photoswitches are electronically connected and display a red-shifted absorption spectrum. The absorption spectrum suggests significant overlap between the two switches, with DHP exhibiting a higher molar extinction coefficient. Consequently, the absorption spectrum of 1 is primarily dominated by the π–π* transitions of DHP. To selectively induce the DHP → CPD isomerization, while leaving the azobenzene unit unaltered, 1 was irradiated with 525 nm visible light. This resulted in a significant hypsochromic shift in the UV/vis spectrum, indicating the opening of the highly conjugated aromatic ring (DHP/*E* → CPD/*E*) (Fig. 4a and b). The quantum yield (*f*) was 1.6 × 10^−4^ (*f*_525nm_) for the ring-opening photoisomerization, which is quite low, as it took approximately 107 min to reach the corresponding PSS (Fig. S27 and Table S1†). Also, a challenge arises during the reversal of this isomerization, as UV light not only reverses the CPD → DHP isomerization but also simultaneously triggers the *E* → *Z* isomerization of azobenzene (Fig. 4c and S24c†). However, selective reversal of the CPD → DHP isomerization can be achieved through thermal treatment, which preserves the *E*-isomer of azobenzene. However, to fully reverse the CPD system, approximately 40 minutes of heating was required for a 10 μM solution in CH_3_CN at 70 °C (Fig. S22†). While effective, thermal treatment is an invasive stimulus and requires a prolonged duration, making it impractical for many real-world applications. Thus, alternative, faster, and less-invasive methods are needed to improve the efficiency and applicability of the system. To address these challenges and develop an orthogonally operating, all-photon photochromic hybrid system, we revisited the literature. Royal and coworkers recently demonstrated that the incorporation of strong electron-withdrawing groups results in a significant redshift in the absorption spectrum, enabling blue-light-induced ring closure (CPD → DHP).^71,72^ To selectively revert the open CPD ring to its closed DHP form in a DHP–azobenzene-coupled system, it is essential to achieve visible-light-mediated CPD → DHP ring-closing. The details of the design principle of the hybrid switch are discussed below. To select a suitable DHP switch, a model derivative, DHPPyMe^+^, was synthesized with Br^−^ as a counter anion. In comparison to the parent DHP, in CH_3_CN, DHPPyMe^+^ exhibited a significantly red-shifted UV/vis absorption spectrum with distinct π–π* bands around 339 nm (*ε* = 71 600 M^−1^ cm^−1^), 415 nm (*ε* = 34 000 M^−1^ cm^−1^), 505 nm (*ε* = 11 100 M^−1^ cm^−1^), and 665 nm (*ε* = 3200 M^−1^ cm^−1^). DHPPyMe^+^ can be photoisomerized with red light (640 nm), and excitation with 640 nm light in a degassed solution of CH_3_CN (10 μM) yielded the corresponding open isomer quantitatively (Fig. 5a and b). The strong absorption bands in the visible region disappeared, giving a colorless solution, a signature of negative photochromism. To determine a suitable visible light wavelength for reversing the transition to the open isomer, different light sources were tested. Although 254 nm UV light achieved the best PSS (89%), enriching DHPPyMe^+^, irradiation with 456 nm light also significantly enriched the closed isomer, reaching a PSS of 76% (Fig. S23†). The visible light (456 nm)-mediated reversion of the open isomer to DHPPyMe^+^ was further confirmed by ^1^H NMR spectroscopy (Fig. S24a†). This suggests that 456 nm light can be employed for DHP ring-closing in systems containing strong electron-withdrawing groups, such as the pyridinium cation. To understand the effect of 456 nm and 640 nm light on azobenzene, we synthesized a control azobenzene derivative, AzoPyMe^+^. Irradiation at 525 nm and 456 nm induced minor *E* → *Z* isomerization of the azobenzene unit, whereas 640 nm light did not cause any isomerization, as confirmed by control experiments using azobenzene alone (Fig. 5c, d and S24b†). Therefore, considering all these points, we synthesized modified DHP- and azobenzene-coupled photochromic dyad derivative 2 with the strategic inclusion of a cationic pyridinium moiety (see ESI† for synthetic details). As DHP has a high molar extinction coefficient, the absorption spectrum is primarily dominated by the π–π* bands of DHP. The UV/vis spectrum of 2 exposed four distinct absorption bands at around 340 nm (*ε* = 77 600 M^−1^ cm^−1^), 425 nm (*ε* = 28 500 M^−1^ cm^−1^), 525 nm (*ε* = 9100 M^−1^ cm^−1^), and 670 nm (*ε* = 2800 M^−1^ cm^−1^) in 10 μM CH_3_CN. Irradiation with 640 nm light led to a significant blue-shift of the absorption bands, accompanied by an increase in the band intensity in the shorter wavelength region (*ca.* 270 nm), consistent with the ring-opening (DHP → CPD) photoisomerization of the DHP unit (Fig. 6a). The reduction in the band intensity in the longer-wavelength region is attributed to the disruption of the highly conjugated aromatic 14 π-electron system into two discrete 6π-electron systems. Visually, the deep-orange-colored solution bleached into a yellowish solution (Fig. 6h). The selective ring opening of DHP from DHP/*E* (State I) → CPD/*E* (State II) was confirmed from the ^1^H-NMR isomerization measurements in CD_3_CN. In DHP/*E*, DHP in its closed isomeric form exhibits a characteristic chemical shift of around −3.87 ppm for the internal methyl protons; the disruption of the aromatic DHP to CPD led these protons to shift to +1.4 ppm, accompanied by a significant shift of the *t*-Bu and other aromatic protons (Fig. 6g). The methoxy (OMe) protons were unaffected, indicating quantitative ring opening without affecting azobenzene. To probe whether the 456 nm light-enabled reversion to DHP could be retained in a coupled system to achieve CPD/*E* → DHP/*E* reversal, we subjected the sample to 456 nm light irradiation on the CPD/*E* (State II) isomer. Exposure for 3 s led to the reappearance of the characteristic UV/vis absorption bands of the closed isomer, suggesting ring closing of the DHP unit (Fig. 6a). The selective ring closing (CPD/*E* → DHP/*E*) is accompanied by the return of the orange color of the solution (Fig. 6h). However, it is important to note that 456 nm light caused a minor degree of *E* → *Z* isomerization of the azobenzene unit, likely due to the redshift in the UV/vis spectrum. ^1^H NMR spectroscopy confirmed the selective CPD → DHP isomerization, with the reappearance of the −3.87 ppm peak along with a small peak at 3.70 ppm, which correspond to the methoxy protons of the *Z* isomer, indicating some degree (30%) of *E* → *Z* conversion of the azobenzene unit (Fig. 6g). This observation is in accordance with the reference derivative AzoPyMe^+^, which showed minor *E* → *Z* isomerization under 456 nm irradiation (Fig. 5d). Subsequent irradiation of the CPD/*E* isomer (State II) with 370 nm light led to the reverse CPD → DHP isomerization of the DHP unit accompanied by the *E* → *Z* isomerization of the azobenzene (State II → State III). The reduced intensity of the UV/vis absorbance band (*ca.* 370 nm) is observed because the π–π* absorption band of azobenzene also resides in this region, and upon *E* → *Z* isomerization, the absorbance of this band decreases and as a whole, reduced intensity is observed (Fig. 6b). The orange color of the solution also returns. The major changes observed in the ^1^H NMR spectrum include a shift of the –OMe protons from 3.86 ppm to 3.70 ppm, indicating *E* → *Z* isomerization, along with previously described changes associated with the CPD → DHP isomerization (Fig. 6g). Additionally, back-to-back irradiation with 456 nm light for 3 s and 640 nm light for 15 s gave the CPD/*E* isomer (State II) following the path State III → State I → State II. Excitation of the DHP/*Z* isomer (State III) with 456 nm light for 3 s resulted in spectral changes in the shorter wavelength region only, suggesting the *Z* → *E* isomerization of the azobenzene without affecting the DHP, thereby directly accessing State I from State III (Fig. 6b). Alternatively, irradiation at 370 nm of the closed-*trans* DHP/*E* isomer (State I) for 3 s led to a decrease in absorbance at *λ* = 370 nm, indicating the reversion of the azobenzene to the *Z* isomer (*E* → *Z*) (State I → State III) (Fig. 6d). ^1^H NMR isomerization also revealed the shift in the signals of methoxy (–OMe) protons, while keeping the other protons associated with DHP intact, indicating *E* → *Z* isomerization (Fig. 6g). Therefore, upon irradiation of the DHP/*Z* isomer with 640 nm light (State III), the characteristic bands of DHP gradually disappear, indicating DHP → CPD ring-opening while keeping the azobenzene in its *E*/*Z*-PSS intact, thereby giving rise to State IV, which is enriched in the CPD/*Z* isomer. Subsequent irradiation of State IV with 370 nm light results in the recovery of State III, as the majority of azobenzene units are already in the Z-form; only the DHP unit undergoes CPD → DHP isomerization (Fig. 6c). The changes were further examined using ^1^H NMR spectroscopy, and the corresponding PSS compositions were evaluated (Fig. 6g). State I can be recovered from State IV; subsequent irradiation of State IV at 456 nm led to the isomerization of both the switches, DHP (CPD → DHP) and azobenzene (*Z* → *E*) (State IV → State I). The UV/vis spectral changes are consistent with the characteristic bands of DHP, and the increase of the absorbance centered at around 370 nm compared to the DHP/*Z* isomer (State III) indicates the *Z* → *E* isomerization of azobenzene (Fig. 6e). The result obtained through irradiation of State IV at 456 nm is also consistent with the State III → State I or State II → State I transformations (Fig. 6e and d). Similarly, another possibility is State IV → State I → State III (Fig. 6e). To showcase the sequential generation of multiple states, we tried generating each state sequentially by changing the light source only in one go: the sequential and consecutive irradiation of 640 nm → 370 nm → 640 nm → 456 nm light generated the four different isomeric states and eventually reverted to the initial state, in the order State I → State II → State III → State IV → State I (Fig. 6f). Therefore, the strategically designed DHP–azobenzene-coupled photochromic systems can be orthogonally switched and used in advanced devices for multi-responsive operations despite having a large overlap in the UV/vis spectrum of the operating region (Fig. 6i). The above system has a few drawbacks, which can be described as follows. (a) The ring-opening quantum yield for the DHP unit using 640 nm light is low (Fig. S27 and Table S1†). Therefore, a 10 μM solution in CH_3_CN requires almost 15 s for DHP → CPD isomerization. (b) The photochemical ring opening under aerobic conditions leads to the formation of unwanted endoperoxide derivatives, thereby decreasing the efficiency of the hybrid switching cycles. During fatigue resistance studies of derivative 2, we found that whenever there was a switching step involving DHP ↔ CPD isomerization, a gradual decay in photostability was observed (Fig. S25†). We were also able to identify the formation of the corresponding endoperoxide for derivative 2 using high-resolution mass spectrometry (HRMS). The experimentally observed pattern matched the simulated pattern (Fig. S21†), consistent with the literature reports of endoperoxides of DHP pyridinium salts.^64,65^ To overcome the disadvantages faced by 2, we synthesized benzo[*e*]-fused dimethyldihydropyrene (BDHP), which switches much faster with better efficiency and also does not react with O_2_, avoiding any endoperoxide formation or degradation even after multiple cycles; hence, flawless switching of BDHP in aerobic conditions was expected.^64^ The benzo[*e*]-fused dimethyldihydropyrene was synthesized following the literature,^73^ and subsequently, 3 was synthesized as its Br^−^ salt following steps similar to those used to obtain 2 (see the ESI† for more details). The tail of the absorbance of 3 extends up to ∼750 nm (Fig. 7a). Unlike derivative 2, the π–π* transition of the azobenzene moiety makes a significant contribution to the UV/vis spectrum of 3. This increased contribution becomes evident during the switching process, in which significant absorbance changes are observed due to the *E*/*Z* isomerization of azobenzene. The transition from State I to State II for 2, corresponding to the DHP → CPD ring-opening, took approximately 15 s under irradiation with 640 nm light in a 10 μM CH_3_CN solution. In contrast, for 3, this process is significantly accelerated, requiring only 3 s of irradiation, indicating a marked enhancement in the photoisomerization quantum yield. Notably, the absorbance tail of 3 extends to nearly 750 nm, enabling ring opening (State Ia → State IIa) using a 740 nm light source. However, due to the significantly reduced molar extinction coefficient at 740 nm, the process requires approximately 15 s (Fig. 7a). The remaining UV/vis isomerization studies align with our previous findings for derivative 2 and follow the same trend. Multiple switching cycles for each state were performed, and the isomerization showed little-to-no decay in photochromic efficiency, which demonstrated the robustness and the fatigue-resistant properties of 3 (Fig. S26†). The PSS distributions of each step were further evaluated by ^1^H NMR isomerization studies (Fig. 7b). The PSS distribution for each of the states improved for 3 compared to 2 (Table 1), which is attributed to the redshift of the BDHP chromophore compared to the parent DHP, leading to better decoupling with the azobenzene spectrum. Both DHP/BDHP and azobenzene belong to the category of T-type photoswitches, *i.e.*, the photogenerated species can return to the stable isomer under heating or in the dark depending on the activation energy (*E*_a_) of the reaction. Therefore, to investigate the stability of the photogenerated states, we performed variable-temperature studies for derivative 3 (Fig. S28, S29 and S31†). Kinetic measurements revealed that the *E*_a_ for BCPD → BDHP is 19.8 ± 0.5 kcal mol^−1^ and the half-life (*t*_1/2_) is 9.5 h; for *Z* → *E* thermal reversal, the *E*_a_ is 25.3 ± 0.5 kcal mol^−1^ and the *t*_1/2_ is almost 53 h (Fig. S30, S32 and Table S2†). The above results suggest that the photogenerated states are highly stable, which is a necessary criterion for using these photo-addressable states. Both derivatives 2 and 3 were found to be water-soluble with the Br^−^ counter anion, and thus, we investigated their photochromic properties in an aqueous medium. Measurement of the photoswitching properties of 3 in water (10 μM) using UV/vis spectroscopy revealed that all four states can be achieved without any significant decay in the photochromic efficiency (Fig. 7c). For almost all types of device applications, it is essential to have photoswitching ability under solvent-free conditions. We monitored the light-mediated absorbance changes in a drop-cast thin film and compared it with the switching in solution (CH_3_CN) (Fig. 7d and S33†). The comparable absorbance spectra of each state suggest that the switching properties were retained in the thin film. Thus, this work presents the development of a multistate addressable photochromic dyad that is robust and compatible with aqueous media and thin films for smart devices or practical applications (Fig. 7e). To explore the possibility of a molecular logic gate based on ATR-FTIR signal output, the FTIR spectra of derivative 3 and all the light-generated states were recorded (Fig. 8a), and the spectrum bands were analyzed to identify the signals that undergo change upon isomerization. The FTIR spectra were recorded on drop-cast and dried samples. The light-gated forward and reverse switching uses two different wavelengths of light as the input and the acquired FTIR bands as the output, which can be utilized to formulate a logic gate. The bands at 1537 cm^−1^ and 1140 cm^−1^ exhibited state-specific vibrations. Derivative 3 in its pristine state—when no light was applied (input *A* = 0 and input *B* = 0)—exhibited no band at 1537 cm^−1^, but showed a signal at 1140 cm^−1^, corresponding to State Ia (pure BDHP/*E* form). Upon the interconversion to State IIa (BCPD/*E*) with 640 nm light (*i.e.*, inputs are *A* = 1 and *B* = 0), a band at 1537 cm^−1^ appeared, and the signal at 1140 cm^−1^ remained unchanged. When we irradiated the sample with only 370 nm wavelength light (*i.e.*, input *A* = 0 and input *B* = 1), we achieved State IIIa (BDHP/*Z*), in which both the signals at 1537 cm^−1^ and 1140 cm^−1^ were absent. Subsequently, upon exposure to both 640 nm and 370 nm light (*i.e.*, when the inputs are *A* = 1 and *B* = 1), it gave another state, State IVa (BCPD/*Z*), and only the vibration signal at 1537 cm^−1^ reappeared, while the band at 1140 cm^−1^ remained less intense. Based on the above analysis of the signals at 1537 cm^−1^ and 1140 cm^−1^, it is clear that the ring opening of the BDHP component (BDHP → BCPD) is responsible for the appearance of the 1537 cm^−1^ signal and the *E* isomeric form of the azobenzene component is responsible for the 1140 cm^−1^ signal. Therefore, the *E* → *Z* isomerization caused the disappearance of the 1140 cm^−1^ peak. These configurations suggest that two different logic gates can be operated. The input–output (I/O) truth table revealed that the information can be represented as YES and NOT B logic gates by monitoring the 1537 cm^−1^ and 1140 cm^−1^ peaks (Fig. 8b).

**Table 1:** PSS achieved upon consecutive irradiation of different wavelengths of light^a^^,^^b^

Compound	PSS at different wavelengths of light, *λ* (nm)				
	Before irradiation	640 nm	370 nm	640 nm	456 nm
2	State I (DHP/*E*)	State I → State II (CPD/*E*)	State II → State III (DHP/*Z*)	State III → State IV (CPD/*Z*)	State IV → State I (DHP/70)
	100 : 100	98/100	72/95	97/92	71 : 70
3	State Ia (BDHP/*E*)	State Ia → State IIa (BCPD/*E*)	State IIa → State IIIa (BDHP/*Z*)	State IIIa → State IVa (BCPD/*Z*)	State IVa → State Ia (BDHP/*E*)
	100 : 100	99/100	81/94	98/93	80 : 71

a PSS: photostationary state.

b The PSS were determined from the ^1^H NMR isomerization studies in 50 mM CD_3_CN solution.

## Conclusions

In summary, we present a novel all-photon stimuli-responsive orthogonal photochromic dyad consisting of a dimethyldihydropyrene-type (DHP/BDHP) and methoxy-substituted azobenzene. Notably, orthogonality could not be achieved by simple coupling of the photoswitches due to the high overlap of the photochemically active regions of the UV/vis spectra. However, a rational structural modification led to a significant bathochromic shift of the absorption spectra, particularly affecting the switching wavelengths of DHP/BDHP, allowing visible light-mediated reversal and ultimately decoupling the activities of the two photoswitches so that they can be switched independently. However, two disadvantages of a pyridinium cation attached to DHP seemed problematic: (a) low photoisomerization quantum yield and (b) O_2_ sensitization, thereby decreasing the photochromic efficiency. These were addressed and solved in derivative 3, in which the photoisomerization quantum yield increased, and the compound was inactive towards O_2_. Therefore, multiple cycles of switching can be achieved, ensuring its fatigue resistance. Interestingly, the substitution of the DHP with the benzo[*e*]-fused dimethyldihydropyrene led to a further redshift of the UV/vis spectra, which enabled NIR light (740 nm)-mediated ring opening (State Ia → State IIa) as well as improved PSS of each of the addressable states. The switching property was demonstrated in thin films as well. Inspired by the results and the robustness of the compound in air and moisture, we explored this dyad system for all-photonic multi-addressable logic applications. The ATR-based FTIR spectrum was used as a non-destructive readout mode for the logic operations. We believe that an easy, cost effective, and non-destructive readout method like ATR-FTIR might open up a plethora of future applications in molecular computing technology.

## Author contributions

S. B. initiated the work by conceptualization, funding acquisition, investigation, methodology, resources, writing, and editing. S. M. performed synthesis, photophysical studies. S. M. and J. A. performed the acquisition of the kinetic data and data analysis. The manuscript was written through the contributions of all authors. All authors have approved the final version of the manuscript.

## Conflicts of interest

There are no conflicts to declare.

## Supplementary Material

SC-016-D5SC02467F-s001

## Data Availability

The data that support the findings of this study are available in the ESI† of this article.
