# Supplementary material for: All-photonic switching of a benzo[e]-fused dimethyldihydropyrene–azobenzene dyad in the solid-state for logic operations
Source: Chem Sci. 2025 Jun 18;16(30):13694–703. doi: 10.1039/d5sc02467f (PMC12223547; doi:10.1039/d5sc02467f)
Supplement: SC-016-D5SC02467F-s001 [file SC-016-D5SC02467F-s001.pdf]

Supplementary Information (SI) for Chemical Science.
This journal is © The Royal Society of Chemistry 2025

Supporting Information for

All-photonic switching of a benzo[e]-fused dimethyldihydropyrene-azobenzene dyad in solid state for logic operations

Sariful Molla, Jakir Ahmed and Subhajit Bandyopadhyay*

Department of Chemical Sciences, Indian Institute of Science Education and Research (IISER) Kolkata, Mohanpur, Nadia 741246, India

* Corresponding author. Email: *sb1@iiserkol.ac.in*

Contents

1. Procedures and Synthesis: 2
   1.1 Materials and methods: 2
   1.2 Photoisomerization studies: 2
   1.3 Synthetic scheme: 3
   1.4 ^{1}H and ^{13}C NMR of the synthesized compounds: 9
   1.5 Mass spectrometry data of the synthesized compounds: 22
2. Thermal reversal of derivative 1: 29
3. Control experiments and selectivity: 30
4. Studies of fatigue resistance: 32
5. Photoisomerization quantum yield: 33
5. Thermal reversal and kinetic studies: 35
   5.1 Arrhenius and Eyring plots: 35

1. Procedures and Synthesis:

1.1 Materials and methods:

All the reagents and solvents in this study were purchased from commercial sources and utilized as supplied unless otherwise indicated. Reactions requiring dry conditions were conducted using oven-dried glassware, and an inert atmosphere was maintained using nitrogen or argon. Standard methods were employed to get the required dry solvents, tetrahydrofuran (THF) was freshly distilled over sodium/benzophenone before use. Acetonitrile (MeCN) and dichloromethane (DCM) were dried by treating with CaH_2 and followed by distillation. Thin-layer chromatography (TLC) was used to monitor all reactions, employing Merck silica gel 60 F254 plates (0.25 mm), visualized under UV light (254 nm) or with appropriate staining. Flash column chromatography was performed using silica gel (Merck, 100-200 mesh), treated with specified solvent systems for each procedure. Solvents were removed using a rotary evaporator at 50°C or lower, and compounds were further dried under high vacuum. Infrared spectra were recorded neat on a Bruker Optics ALPHAE spectrometer with a universal Zn-Se ATR (attenuated total reflection) accessory, given in wavenumbers (cm^{-1}). ^1H and ^{13}C nuclear magnetic resonance (NMR) spectra were recorded on Jeol spectrometer (400 MHz for ^1H and 100 MHz for ^{13}C), or Bruker (500 MHz for ^1H and 125 MHz for ^{13}C). Chemical shifts (δ) are reported in parts per million (ppm) relative to tetramethylsilane (TMS), referenced to residual solvent signals.

1.2 Photoisomerization studies:

The photoisomerization experiments were conducted in acetonitrile at 298 K, unless specified otherwise. Compound **1** underwent closed to open photoisomerization under 525 nm green light, while for **2** and **3** 640 nm red LED light was used. We have consistently maintained a distance of 6 cm between the light source and the sample for our studies. The power of the light sources were 35 mW (for 640 nm) and 137 mW (for 525 nm). The reverse isomerization under different light conditions was studied as described in the figure captions. To minimize the heating effects, the 1 mL cuvette was maintained under ice-cold conditions during the photo-irradiation. UV/Vis absorbance data were recorded using a Cary 60 UV-Vis spectrophotometer. Thermal open to closed isomerization was performed at various temperatures using a Peltier module heating system within the spectrophotometer with an accuracy of ±1 °C.

1.3 Synthetic scheme:

Scheme S1: Synthesis of derivative **1**.

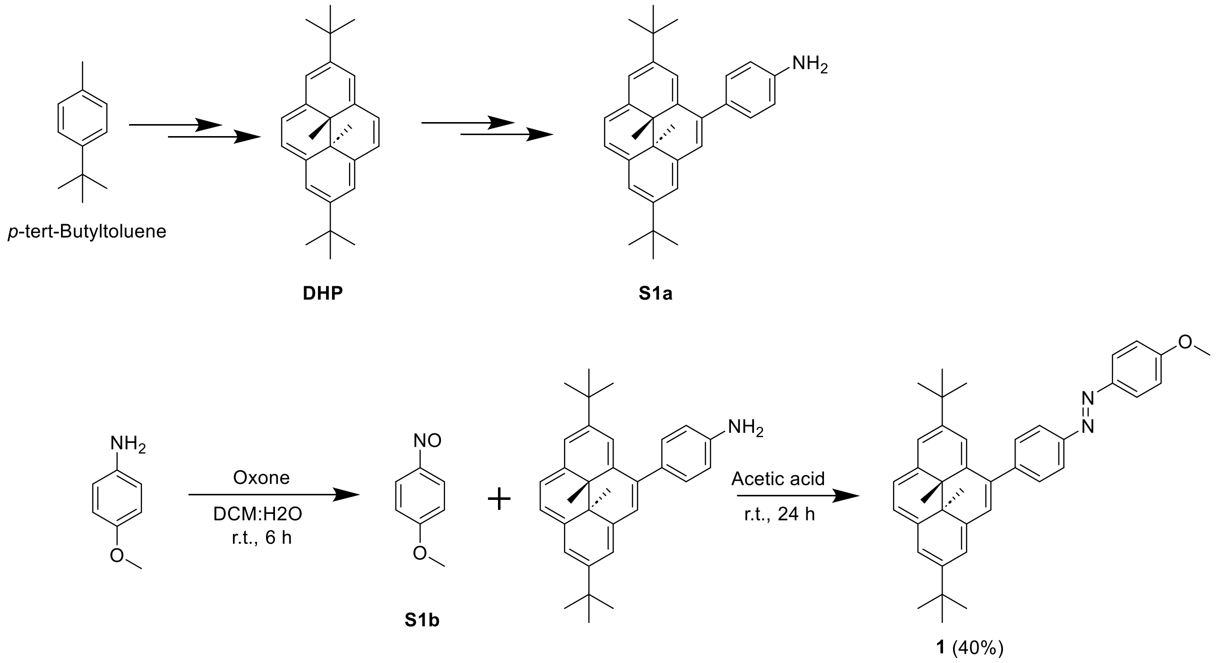

The synthesis of unsubstituted DHP was carried out from 4-*tert*-butyltoluene through a seven-step process, as outlined in the literature.[1] The purity of the synthesized product was confirmed by ^{1}H NMR and ^{13}C NMR spectroscopy, with both spectra showing results that are consistent with previously reported data. Bromination of the DHP was performed according to established methods, yielding mono-substituted DHP-Br, with ^{1}H NMR and ^{13}C NMR data in agreement with literature values.[2] Subsequently, the synthesis of **S1a** was achieved by a Suzuki-Miyaura coupling reaction following the reported procedure.[3]

Additionally, compound **S1b** was synthesized using a previously described procedure.[4]

(*E*)-1-(4-((3a1R,5a1R)-2,7-di-tert-butyl-3a1,5a1-dimethyl-3a1,5a1-dihydropyren-4-yl)phenyl)-2-(4-methoxyphenyl)diazene (1):

S1a (100 mg, 0.23 mmol) was added to a solution of **S1b** (32 mg, 0.23 mmol) and glacial acetic acid (2 mL). the reaction mixture was stirred for 24 h at room temperature. The reaction was monitored by TLC. After completion of the reaction, 25 mL water was added to the reaction mixture and neutralized by adding sat. aq. $NaHCO_3$-solution. Then, the organic compound was extracted with CH_2Cl_2 (3 × 30 mL), and washed with brine (10 mL). The organic layer was dried over anhydrous Na_2SO_4, filtered and concentrated in *vacuo*. The crude product was purified by column chromatography (silica gel, eluent: 5% ethyl acetate in hexane) to give 50 mg of pure compound (yield 40%).

^{1}H NMR (400 MHz, CDCl$_3$) δ: 8.72 (s, 1H), 8.57 (s, 4H), 8.54 (m, 3H), 8.46 (s, 2H), 8.16 – 8.11 (m, 2H), 8.07 – 7.95 (m, 4H), 7.12 – 7.03 (m, 2H), 3.93 (s, 4H), 1.70 (s, 11H), 1.61 (s, 9H), –3.82 (d, *J*=9.0, 6H).

^{13}C NMR (100 MHz, CDCl$_3$) δ: 162.18, 151.61, 147.42, 146.19, 145.39, 137.36, 136.90, 136.40, 134.12, 133.46, 131.90, 128.43, 124.92, 124.79, 123.15, 122.93, 122.62, 121.32, 121.17, 121.00, 120.17, 114.44, 55.77, 36.28, 36.11, 32.08, 31.98, 30.29, 29.85, 22.84, 14.27, 1.17, 0.14.

HRMS (ESI): *m/z* (Calc): $C_{39}H_{42}N_2O[M+H]^+$ 555.3370; found: 555.3375.

Scheme S2: Synthesis of compound **2**.

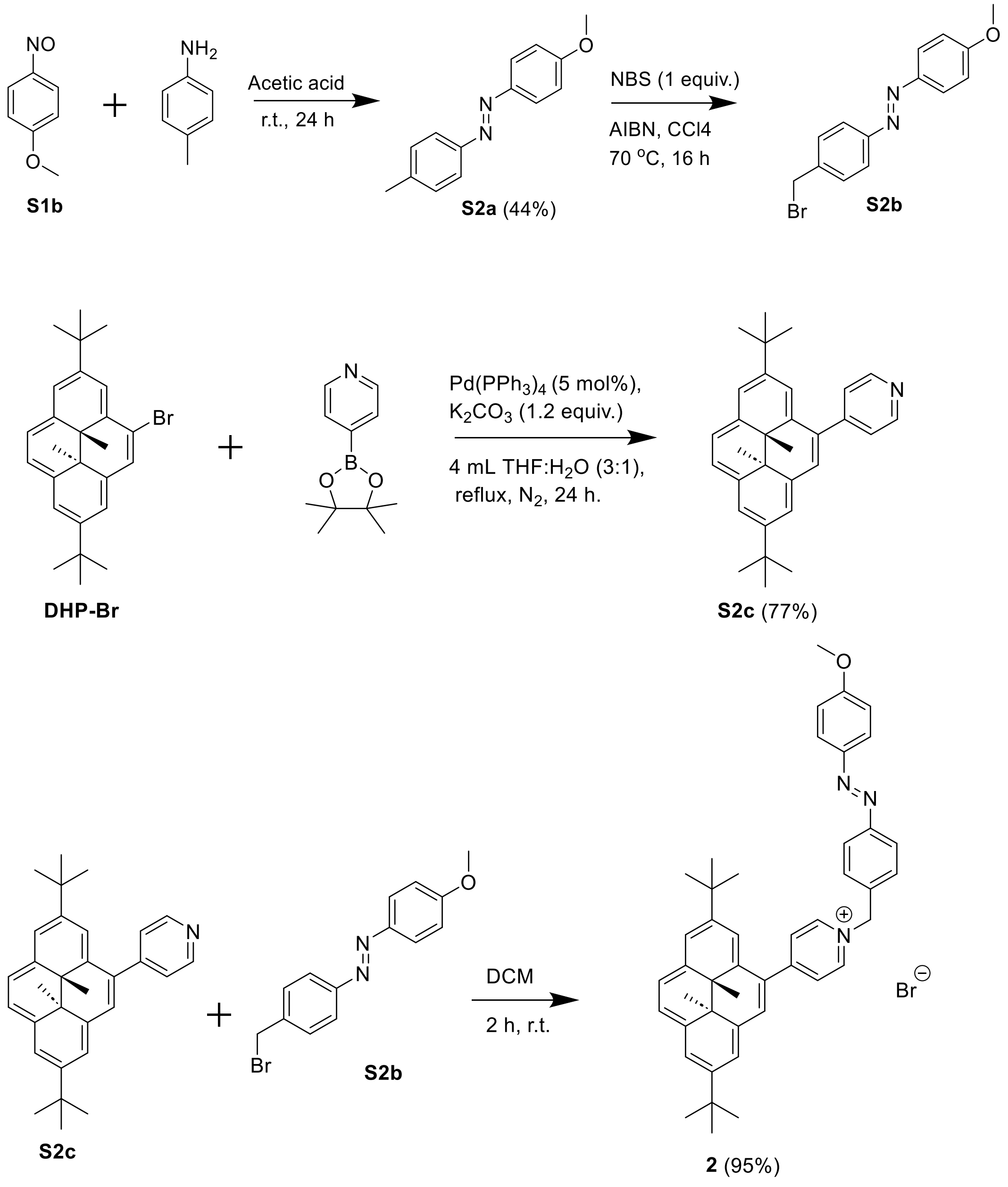

(*E*)-1-(4-methoxyphenyl)-2-(p-tolyl)diazene (S2a):

4-Aminotoluene (500 mg, 4.66 mmol) was added to a solution of **S1b** (640 mg, 4.66 mmol) and glacial acetic acid (5 mL). the reaction mixture was stirred for 24 h at room temperature. The reaction was monitored by TLC. After completion of the reaction, 30 mL water was added to the reaction mixture and neutralized by adding sat. aq. $NaHCO_3$-solution. Then, the organic compound was extracted with CH_2Cl_2 (3 × 30 mL), and washed with brine (10 mL). The organic layer was dried over anhydrous Na_2SO_4, filtered and concentrated in *vacuo*. The crude product was purified by column chromatography (silica gel, eluent: 5% ethyl acetate in hexane). The product obtained as a yellowish powder, (yield 44 %, 464 mg).

m.p: 103-105°C

^{1}H NMR (400 MHz, $CDCl_3$) δ: 7.93 (d, *J*=8.9, 0H), 7.82 (d, *J*=8.6, 0H), 7.31 (d, *J*=8.0, 0H), 7.02 (d, *J*=9.0, 0H), 3.89 (s, 1H), 2.44 (s, 1H).

^{13}C NMR (100 MHz, $CDCl_3$) δ: 162.05, 150.82, 147.08, 141.00, 129.83, 124.81, 122.69, 114.33, 55.68, 21.58.

HRMS (ESI): *m/z* (Calc): $C_{14}H_{14}N_2O[M+H]^+$ 227.1179; found: 227.1198.

(*E*)-1-(4-(bromomethyl)phenyl)-2-(4-methoxyphenyl)diazene (S2b):

To a solution of **S2a** (482 mg, 2 mmol) in CCl_4 (20 mL), NBS (392 mg, 2.2 mmol) and AIBN (33 mg, 0.2 mmol) were added and kept it under reflux for 16h at 70 °C. After bringing down the temperature to room temperature, 20 mL water was added. The organic layer was then extracted with CH_2Cl_2 (3 × 30 mL), and washed with brine (10 mL). The organic mixture was dried over anhydrous Na_2SO_4, filtered and evaporated *in vacuo*. The crude obtained was a mixture and used for the next reaction without further purification.

4-((3a1R,5a1R)-2,7-di-tert-butyl-3a1,5a1-dimethyl-3a1,5a1-dihydropyren-4-yl)pyridine (S2c):

Compound **S2c** was synthesized according to a reported synthetic procedure.[5] And the purity of the compound was confirmed by the ^{1}H NMR spectroscopy.

^{1}H NMR (400 MHz, $CDCl_3$) δ: 8.87 – 8.82 (m, 2H), 8.63 (s, 1H), 8.61 – 8.57 (m, 2H), 8.50 (d, J=2.1, 2H), 8.47 (s, 1H), 7.93 – 7.89 (m, 2H), 1.69 (s, 9H), 1.61 (s, 9H), -3.87 (d, J=9.6, 6H).

^{13}C NMR (100 MHz, $CDCl_3$) δ: 153.13, 147.50, 147.20, 146.71, 137.85, 137.02, 136.30, 133.20, 130.55, 126.69, 123.91, 123.88, 123.66, 122.11, 121.97, 121.59, 118.88, 36.37, 36.15, 32.02, 31.91, 30.35, 29.70, 14.98, 14.57.

4-((3a1R,5a1R)-2,7-di-tert-butyl-3a1,5a1-dimethyl-3a1,5a1-dihydropyren-4-yl)-1-(4-((E)-(4-methoxyphenyl)diazenyl)benzyl)pyridin-1-ium bromide (2):

S2c (50 mg, 0.118 mmol) was dissolved in dichloromethane (5 mL). **S2b** (54 mg, 0.177 mmol) was then added to the solution and kept under stirring at ambient conditions for 2 h. Then the solvent was removed under reduced pressure to obtain the crude. The crude was further washed

with pentane (3 × 10 mL), and dried in *vacuo*. The compound obtained was reddish black in color, (yield 95 %, 80 mg).

^{1}H NMR (400 MHz, CD_3CN) δ: 9.08 – 9.00 (m, 2H), 8.82 – 8.78 (m, 1H), 8.78 – 8.74 (m, 3H), 8.68 – 8.57 (m, 3H), 8.49 (d, J=6.9, 2H), 8.00 (d, J=8.7, 2H), 7.94 (d, J=9.2, 2H), 7.83 – 7.80 (m, 2H), 7.11 (d, J=9.1, 2H), 5.99 (s, 2H), 3.90 (s, 3H), 1.69 (s, 9H), 1.64 (s, 9H), -3.87 (d, J=17.5, 6H).

^{13}C NMR (125 MHz, CD_3CN) δ: 162.86, 159.56, 153.33, 149.23, 147.38, 146.78, 143.78, 139.02, 137.04, 136.12, 135.39, 133.75, 130.43, 129.56, 127.03, 125.07, 124.88, 124.06, 123.68, 123.26, 123.22, 123.16, 122.03, 117.98, 114.58, 62.98, 55.53, 36.16, 35.76, 31.02, 30.86, 30.48, 29.29, 14.48, 14.04.

HRMS (ESI): *m/z* (Calc): $C_{45}H_{48}N_3O^+$[M]$^+$ 646.3792; found: 646.3795.

Scheme S3: Synthesis of derivative **3**.

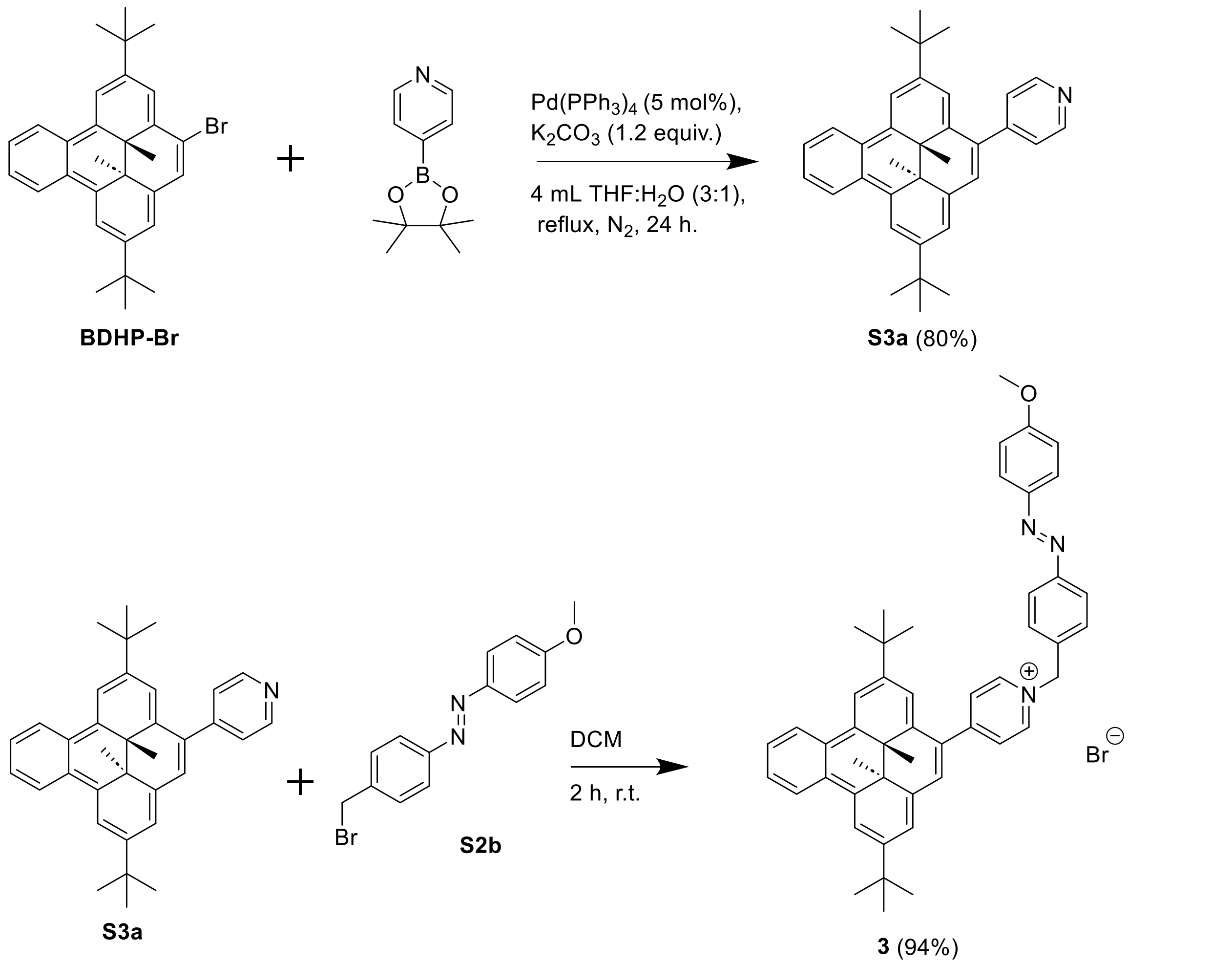

4-((3a1S,5a1S)-2,7-di-tert-butyl-3a1,5a1-dimethyl-3a1,5a1-dihydrobenzo[e]pyren-4-yl)-1-(4-((E)-(4-methoxyphenyl)diazenyl)benzyl)pyridin-1-ium bromide (3):

S3a (50 mg, 0.106 mmol) was dissolved in dichloromethane (5 mL). **S2b** (49 mg, 0.159 mmol) was then added to the solution and kept under stirring at ambient conditions for 2 h. The precipitate formed (bromide salt) was filtered off, washed with diethyl ether (3 × 20 mL), and dried *in vacuo* affording 78 mg (94 %,) of pure compound **3** as a blackish red solid.

m.p: 187−189°C

^{1}H NMR (400 MHz, CD_3CN) δ: 9.14 (d, J=6.4, 2H), 8.96 – 8.87 (m, 2H), 8.44 (d, *J*=20.0, 2H), 8.22 (d, J=6.4, 2H), 7.91 (dd, *J*=12.9, 8.4, 4H), 7.82 (d, *J*=8.2, 2H), 7.72 – 7.67 (m, 3H), 7.48 (s, 1H), 7.30 (s, 1H), 7.07 (d, *J*=8.6, 2H), 6.06 (s, 2H), 3.87 (s, 3H), 1.48 (d, *J*=0.6, 9H), 1.45 (d, J=0.6, 9H), −1.47 (d, *J*=19.7, 6H).

^{13}C NMR (100 MHz, CD_3CN) δ: 162.83, 158.60, 153.22, 150.65, 146.82, 146.78, 144.13, 138.87, 138.75, 137.81, 135.83, 135.05, 130.50, 129.43, 129.02, 128.63, 127.15, 126.73, 126.45, 125.24, 124.91, 124.81, 123.13, 121.13, 119.80, 118.04, 115.13, 114.60, 62.56, 55.58, 54.44, 36.85, 35.99, 35.33, 35.12, 29.74, 29.68, 17.35.

HRMS (ESI): *m/z* (Calc): $C_{49}H_{50}N_3O^+[M]^+$ 696.3949; found: 696.3947.

Scheme S4: Syntheses of compounds **4** and **5**.

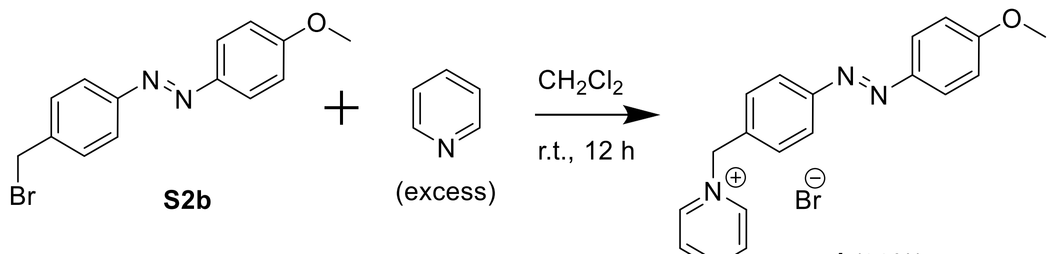

4 (92%)

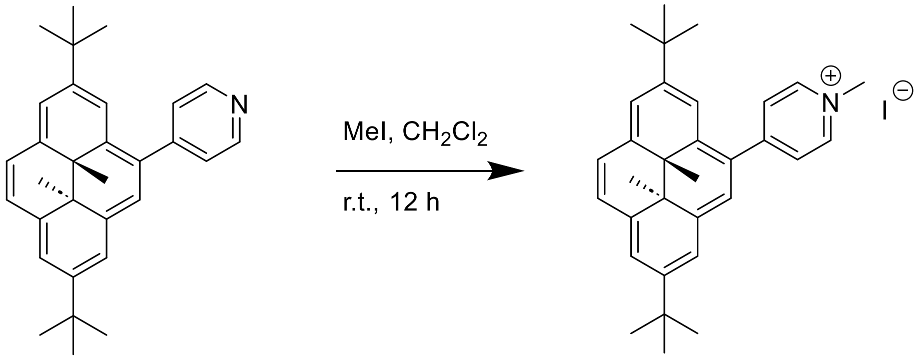

5 (95%)

(*E*)-1-(4-((4-methoxyphenyl)diazenyl)benzyl)pyridin-1-ium (4):

A solution of **S2b** (30 mg, 0.098 mmol) in CH_2Cl_2 (5 mL) was prepared. Pyridine (0.5 mL, excess) was added to it and the resulting mixture was stirred for 12 h at room temperature. The solvent was then evaporated *in vacuo* and the residue was washed with pentane (3 × 10 mL). The resulting solid was dried under vacuum to afford the desired product **4** as a reddish solid (35 mg, 92%).

m.p: 167-169°C

^{1}H NMR (400 MHz, CD_3CN) δ: 9.08 (d, J=5.1, 2H), 8.54 (t, *J*=7.8, 1H), 8.09 – 8.04 (m, 2H), 7.90 (dd, *J*=8.6, 3.9, 4H), 7.71 (d, *J*=8.3, 2H), 7.09 (d, *J*=9.0, 2H), 5.99 (s, 2H), 3.88 (s, 3H).

^{13}C NMR (101 MHz, CD_3CN) δ: 162.87, 153.27, 146.79, 146.28, 144.96, 135.36, 130.47, 128.63, 124.91, 123.10, 114.61, 63.49, 55.58.

HRMS (ESI): *m/z* (Calc): $C_{19}H_{18}N_3O^+[M]^+$ 304.1445; found: 304.1451.

4-((3a1R,5a1R)-2,7-di-tert-butyl-3a1,5a1-dimethyl-3a1,5a1-dihydropyren-4-yl)-1-methylpyridin-1-ium (5):

Methyl iodide (1 mL, excess) was added to a solution of **S2c** (30 mg, 0.071 mmol) in CH_2Cl_2 (5 mL). The resulting mixture was stirred for 12 h at room temperature. The solvent was then removed under vacuum and the residue was washed with diethyl ether (3 × 10 mL). The solid was then dried under reduced pressure to give 38 mg (95%) of pure compound **5** as a brown solid.

^{1}H NMR (400 MHz, CD_3CN) δ: 8.87–8.77 (m, 3H), 8.76 (d, *J*=3.2, 2H), 8.72 (s, 1H), 8.68 – 8.59 (m, 3H), 8.45 (d, *J*=6.7, 2H), 4.44 (s, 3H), 1.69 (s, 9H), 1.64 (s, 9H), -3.88 (d, *J*=16.3, 6H).

^{13}C NMR (100 MHz, CD_3CN) δ: 158.82, 148.97, 147.37, 144.65, 138.80, 137.06, 136.12, 133.59, 129.19, 127.27, 124.97, 124.07, 123.59, 123.16, 123.09, 122.02, 117.99, 47.62, 36.20, 35.83, 31.10, 30.94, 30.40, 29.31, 14.47, 14.02.

HRMS (ESI): *m/z* (Calc): $C_{32}H_{38}N^+[M]^+$ 436.2999; found: 436.2992.

1.4 ^{1}H and ^{13}C NMR of the synthesized compounds:

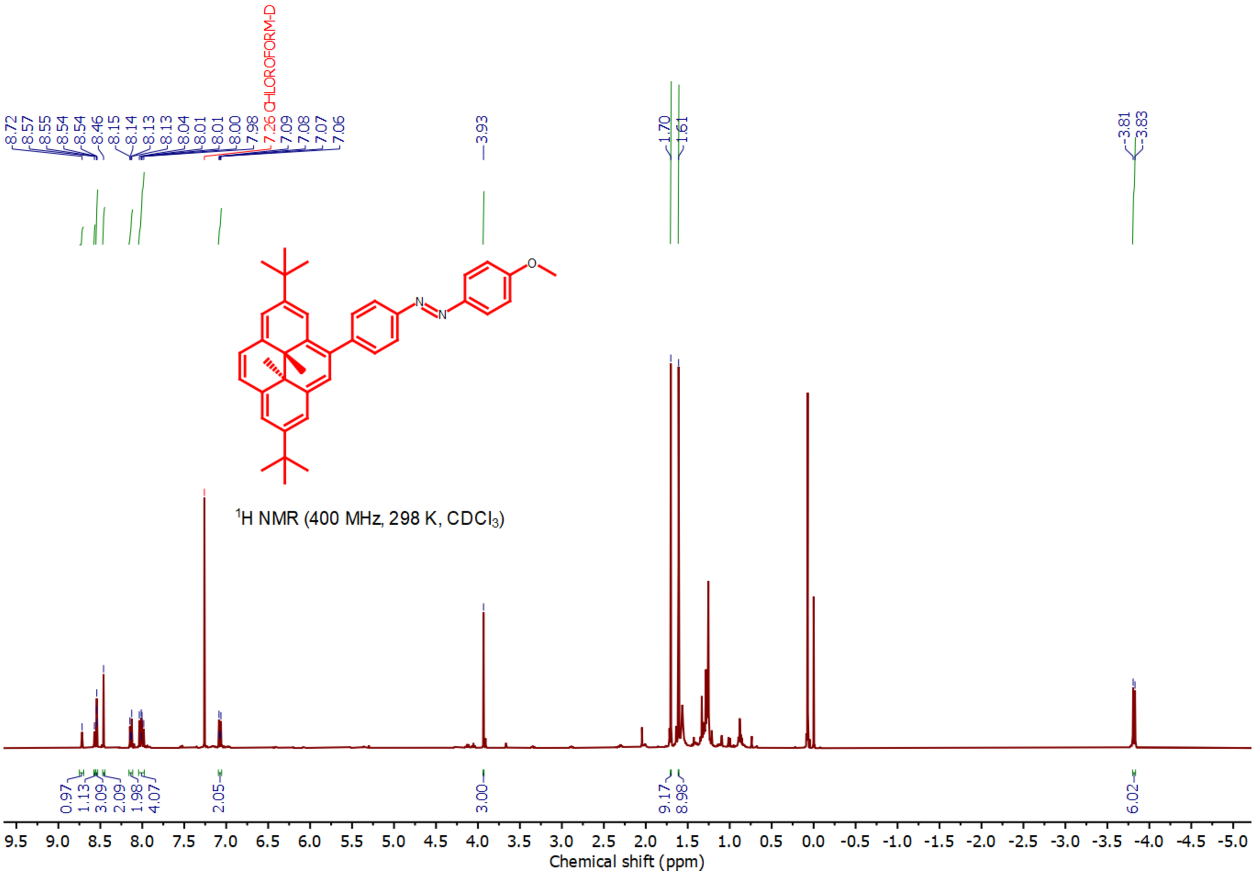

Figure S1: ^{1}H NMR spectrum of **1** in $CDCl_3$.

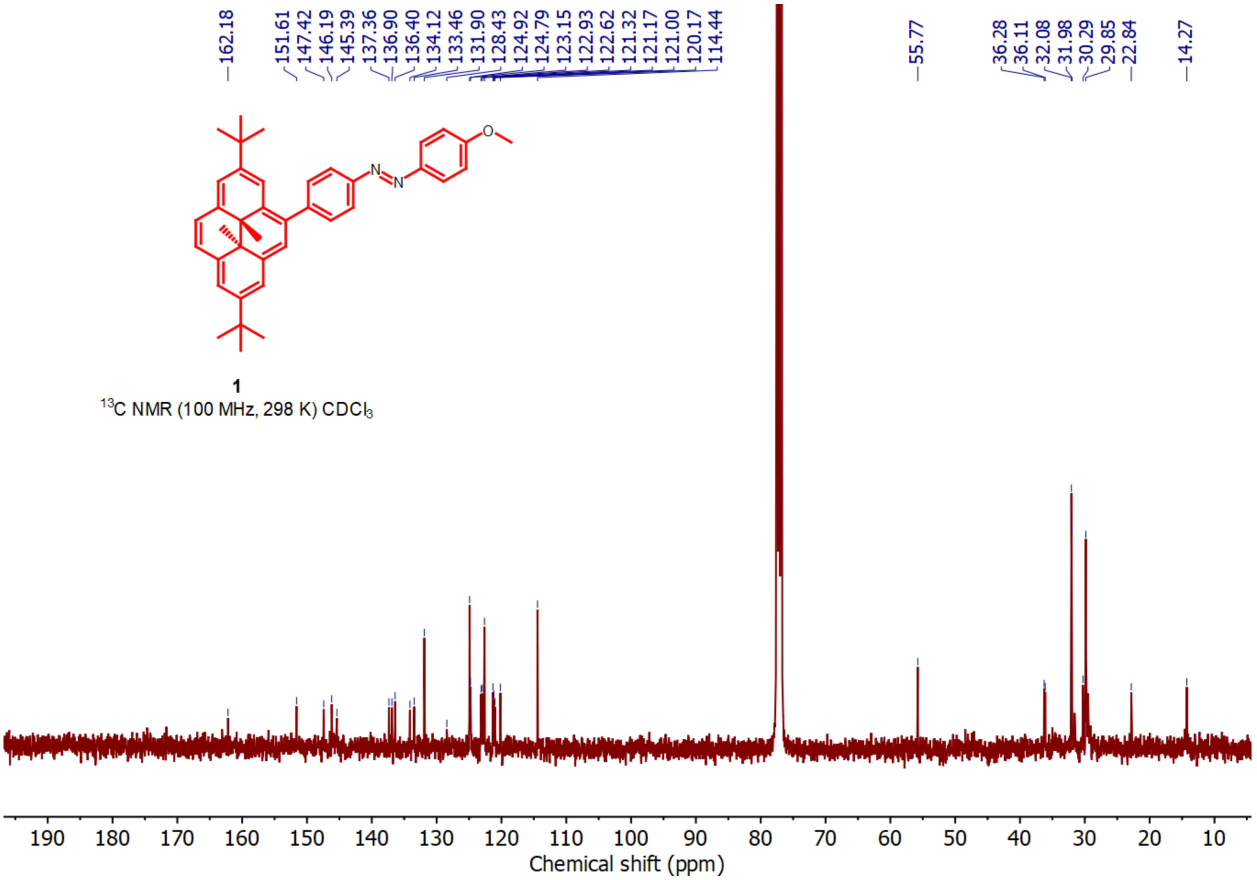

Figure S2: ^{13}C NMR spectrum of **1** in $CDCl_3$.

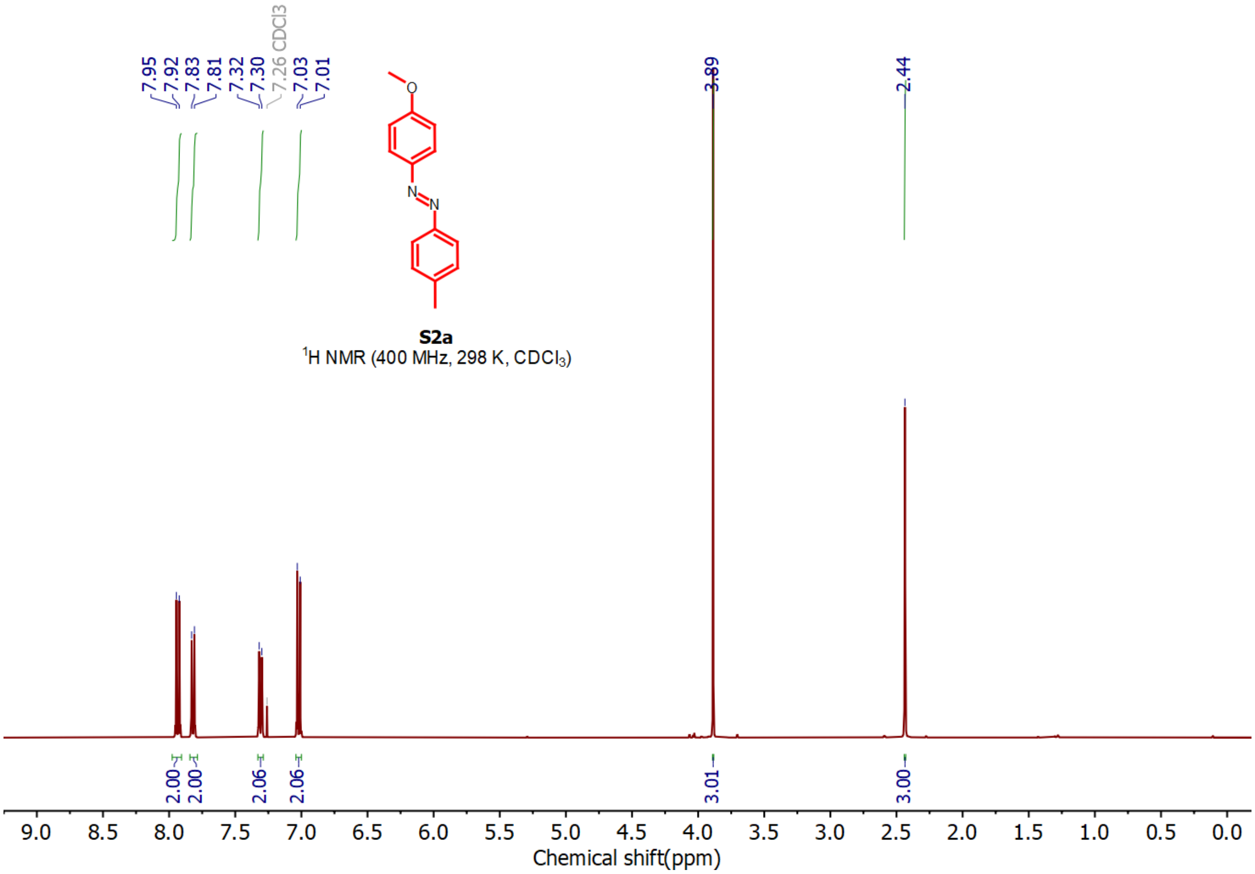

Figure S3: ^{1}H NMR spectrum of **S2a** in $CDCl_3$.

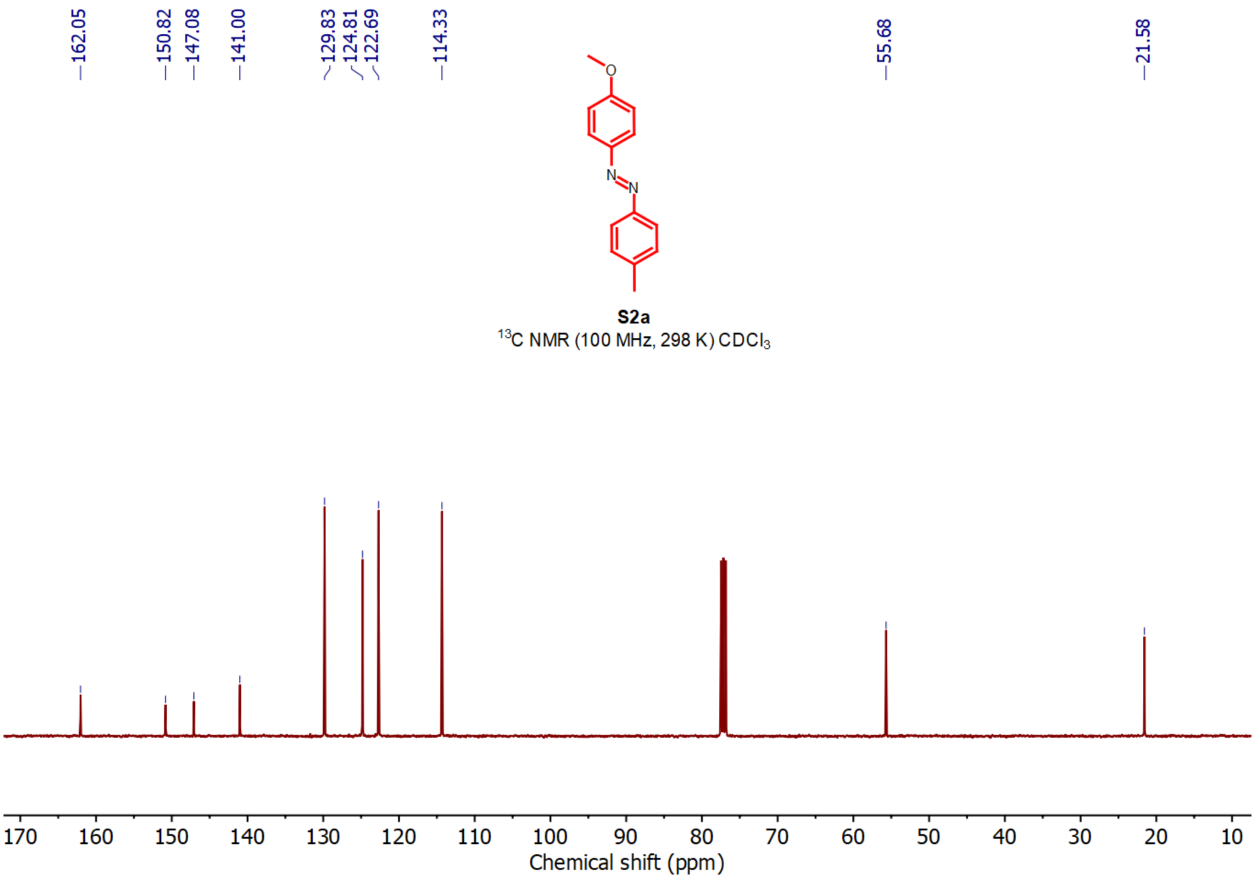

Figure S4: ^{13}C NMR spectrum of **S2a** in $CDCl_3$.

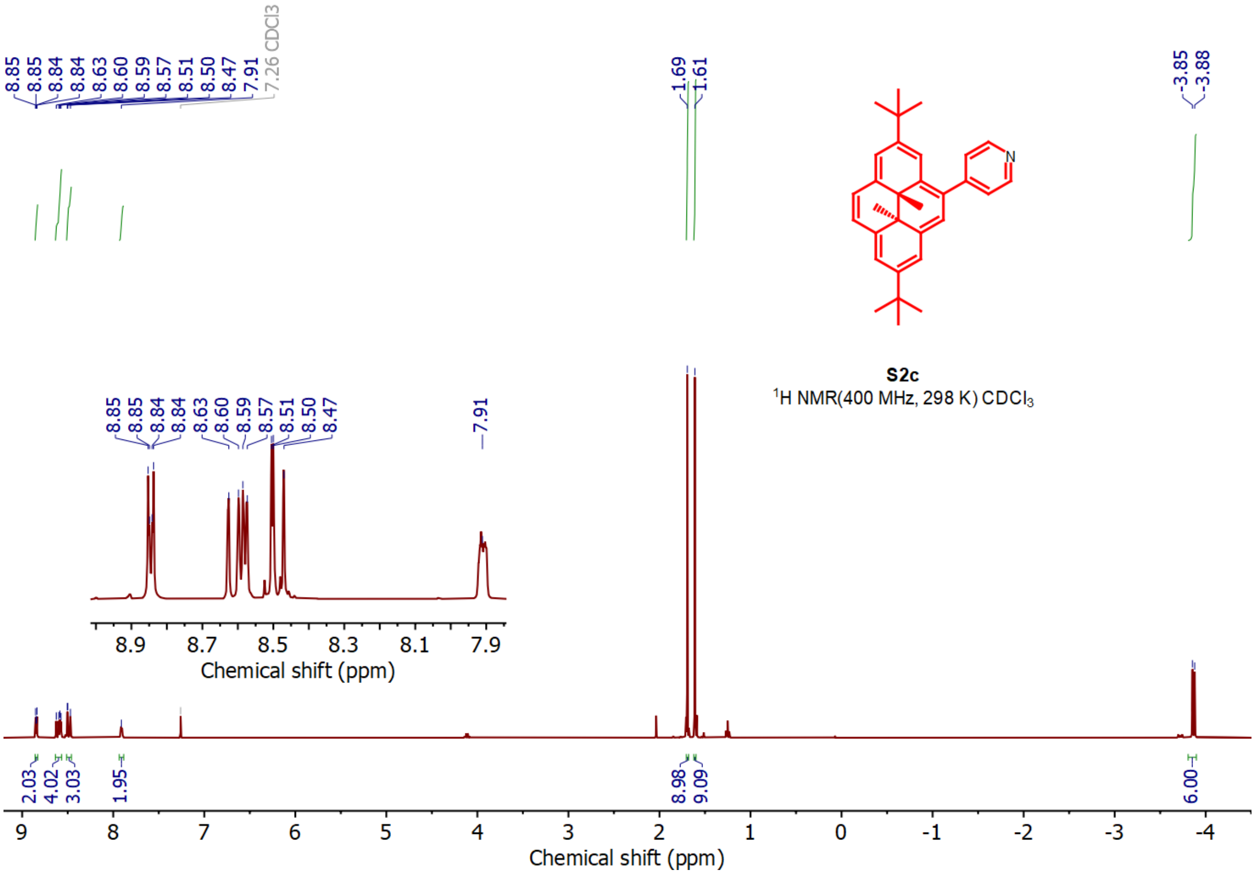

Figure S5: ^{1}H NMR spectrum of **S2c** in $CDCl_3$.

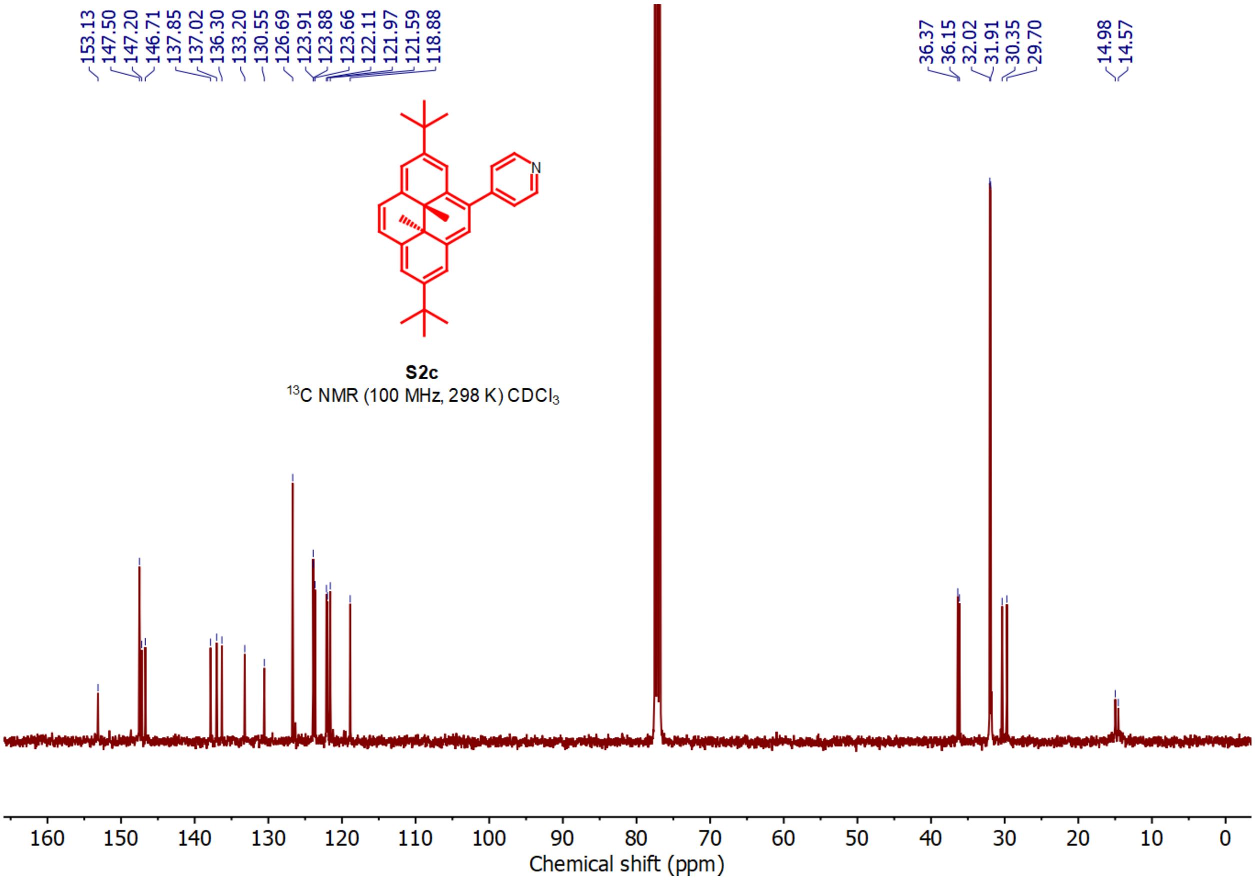

Figure S6: ^{13}C NMR spectrum of **S2c** in $CDCl_3$.

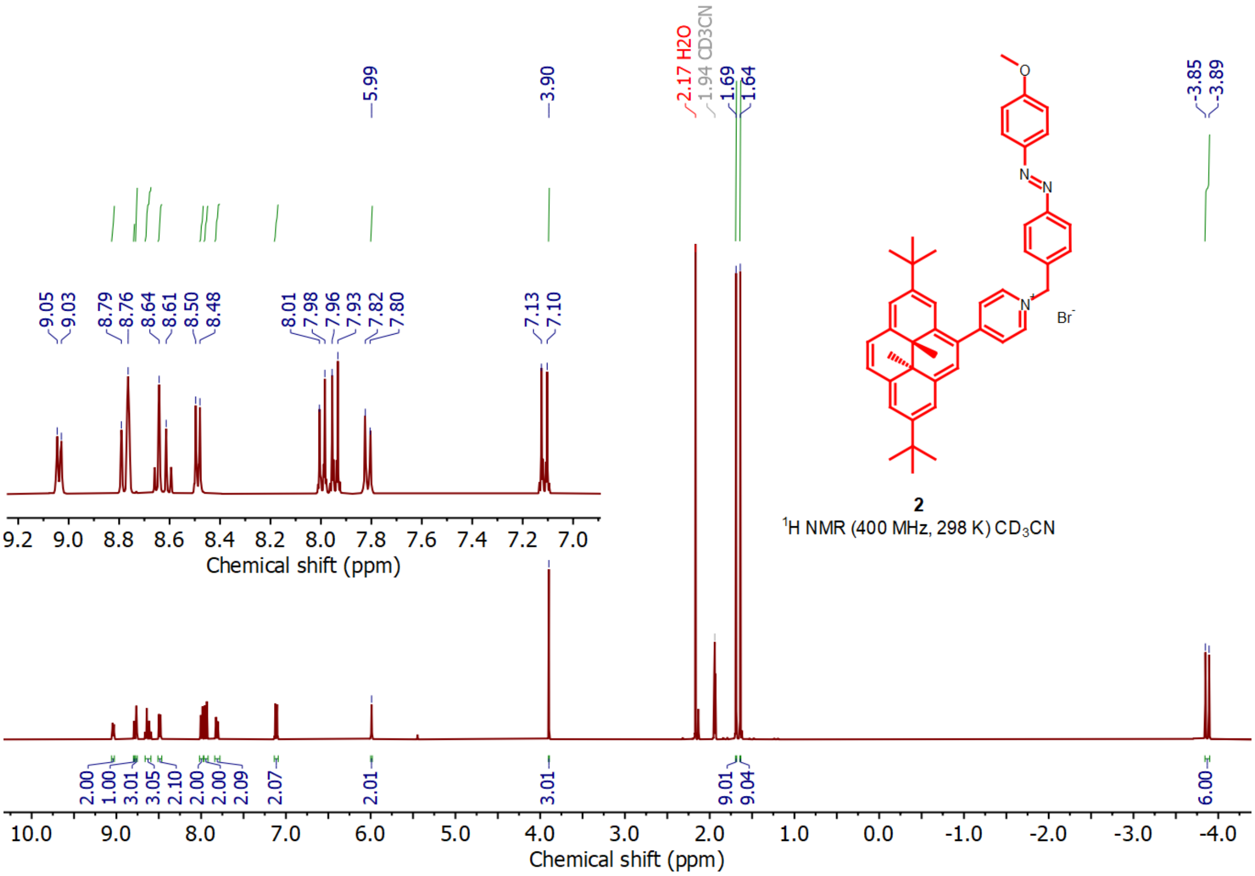

Figure S7: ¹H NMR spectrum of **2** in CD_3CN.

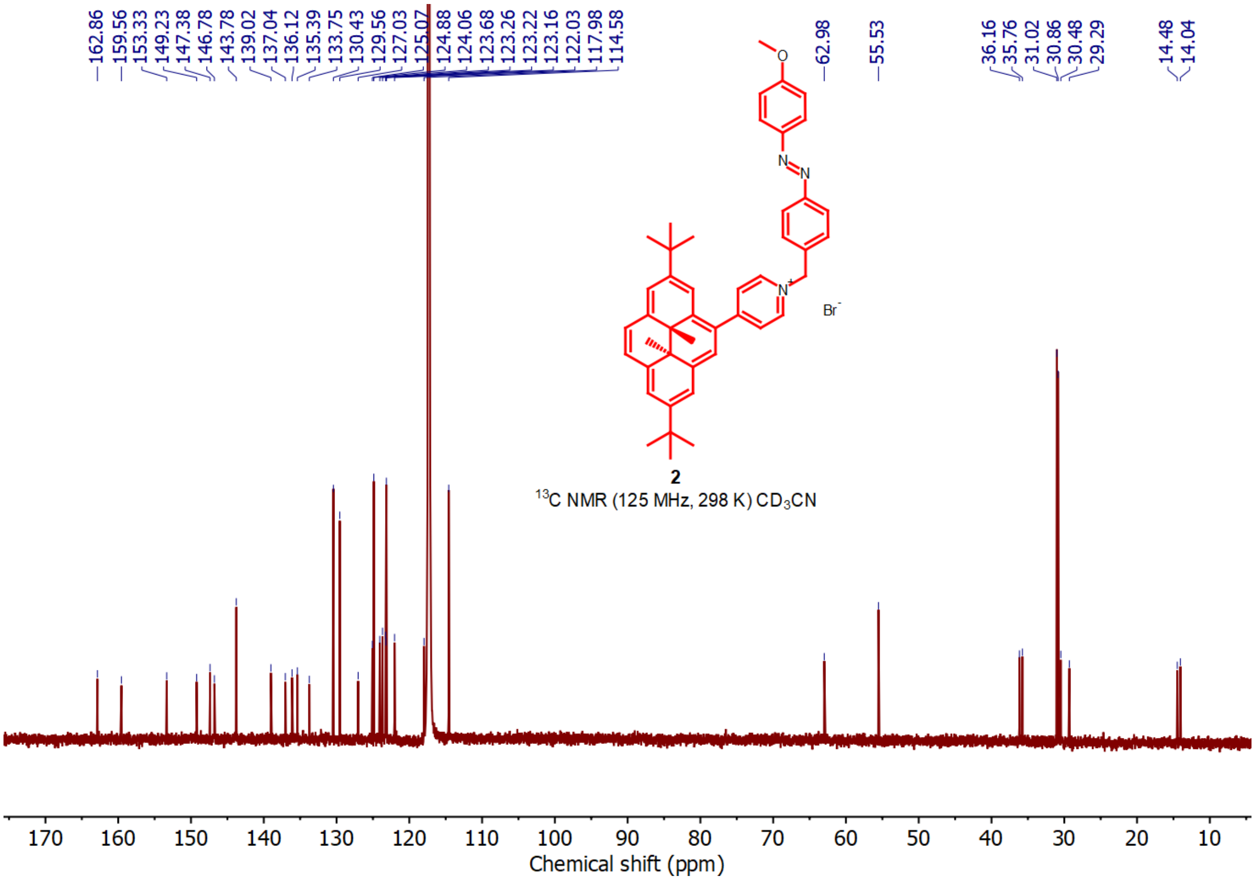

Figure S8: ^{13}C NMR spectrum of **2** in CD_3CN.

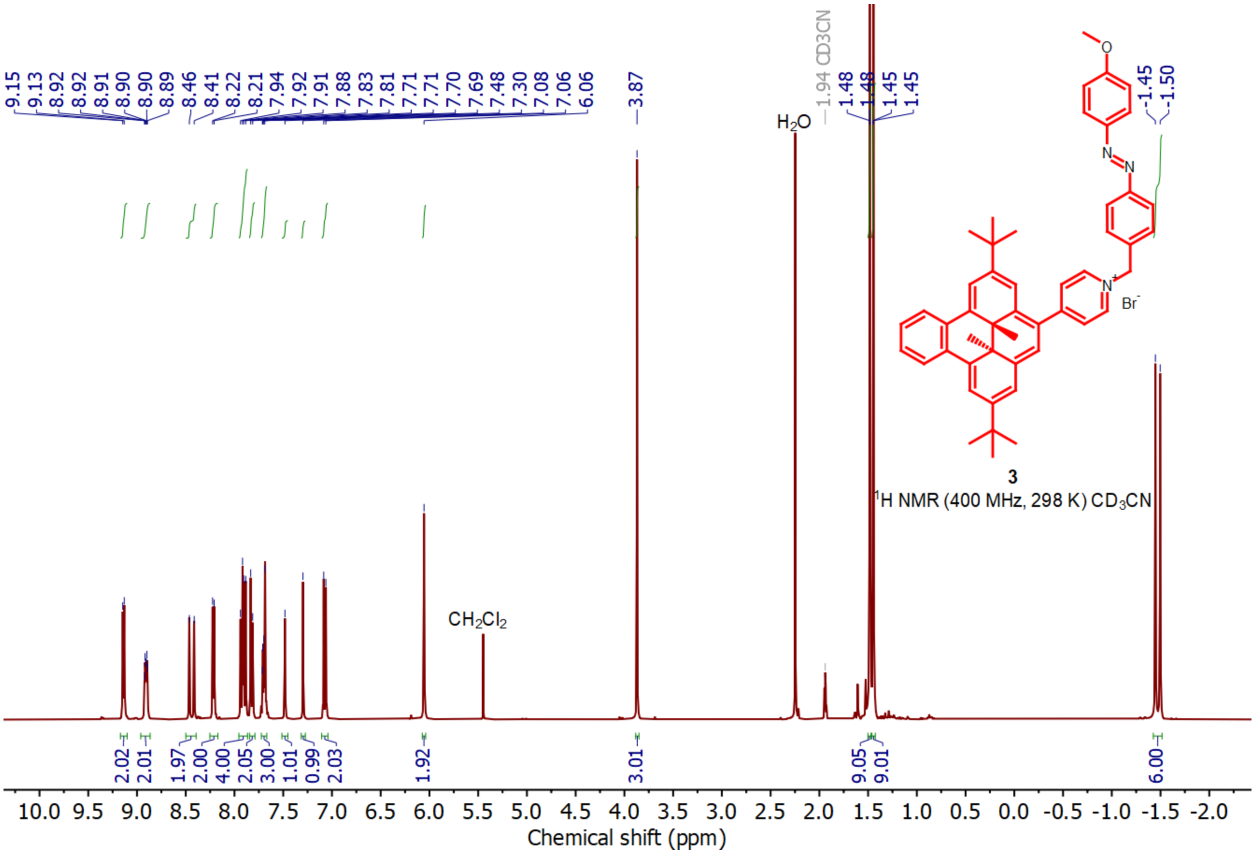

Figure S9: ^{1}H NMR spectrum of **3** in CD_3CN.

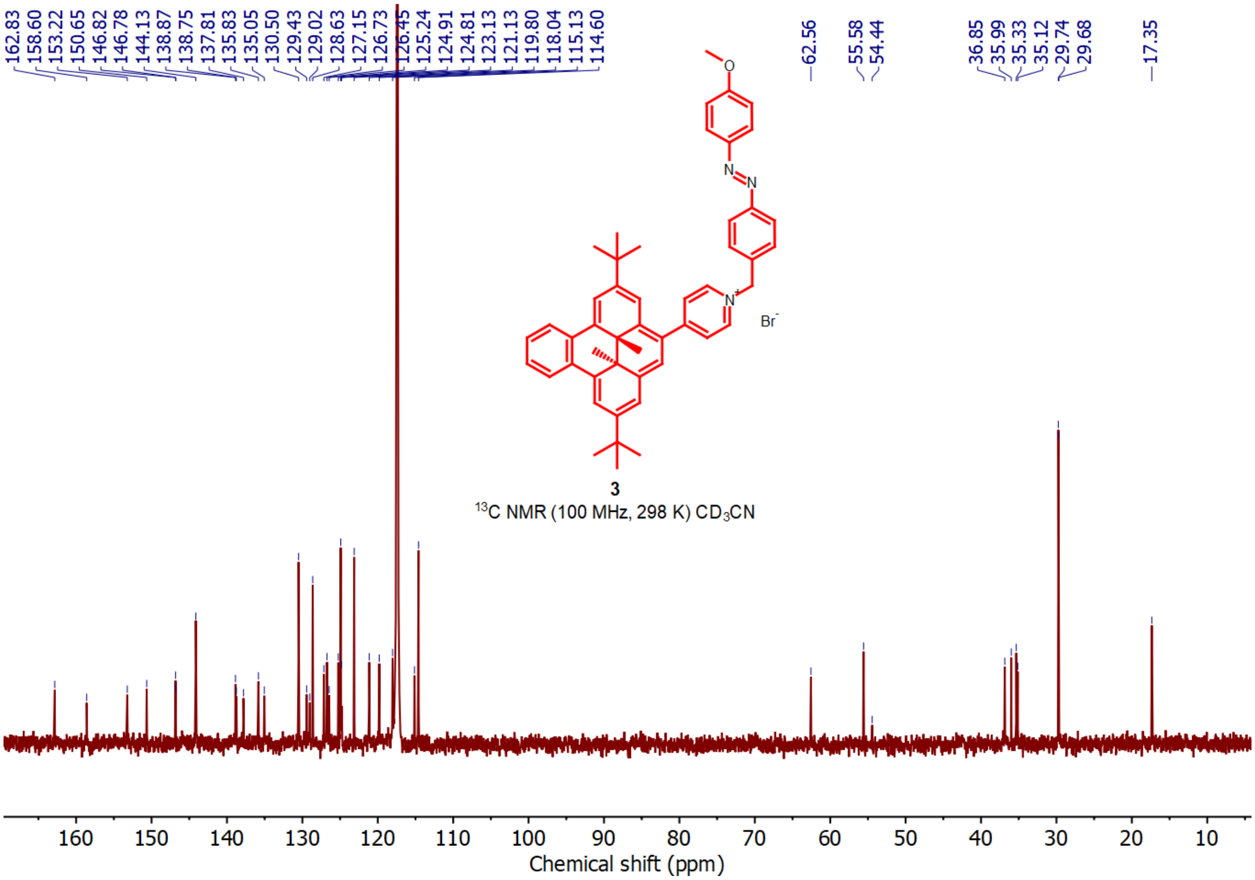

Figure S10: ^{13}C NMR spectrum of **3** in CD_3CN.

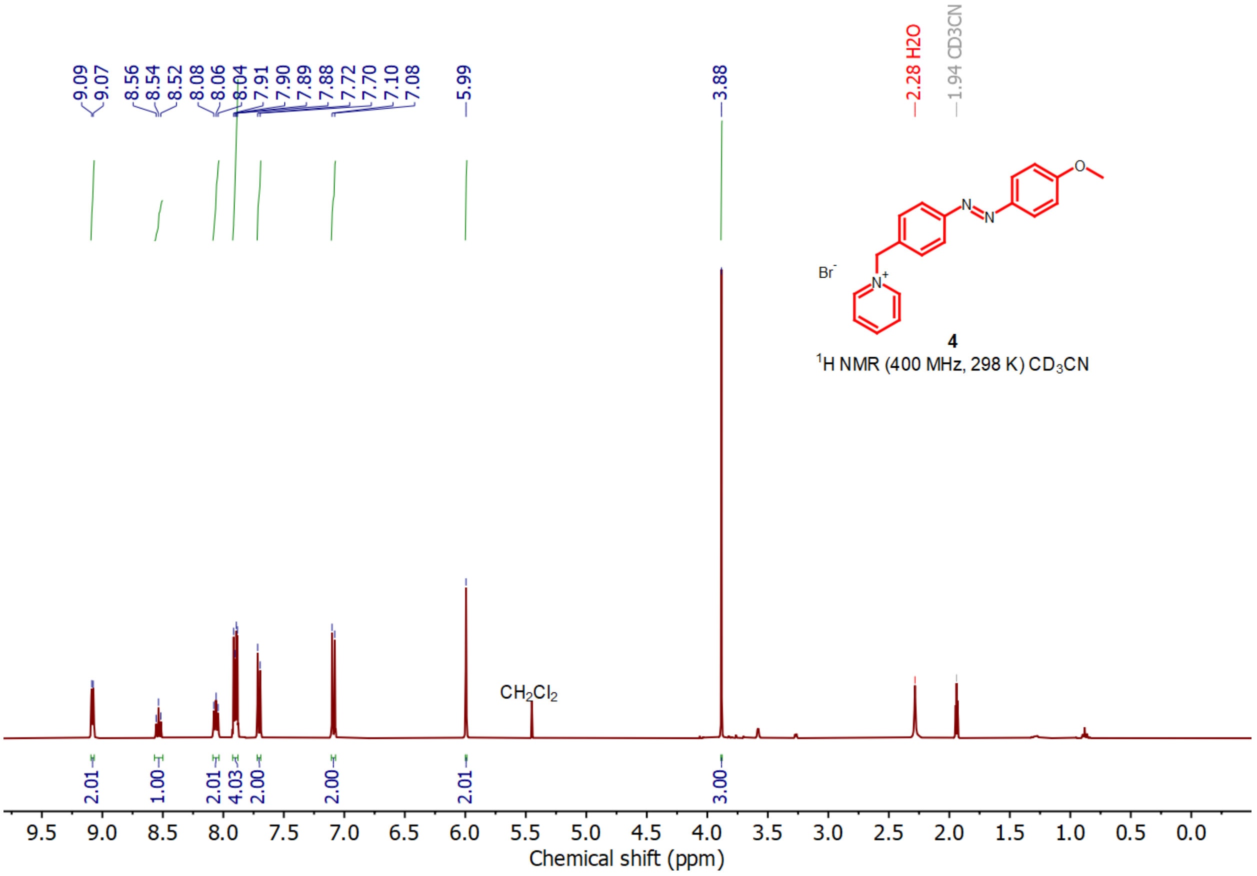

Figure S11: ^{1}H NMR spectrum of **4** in CD_3CN.

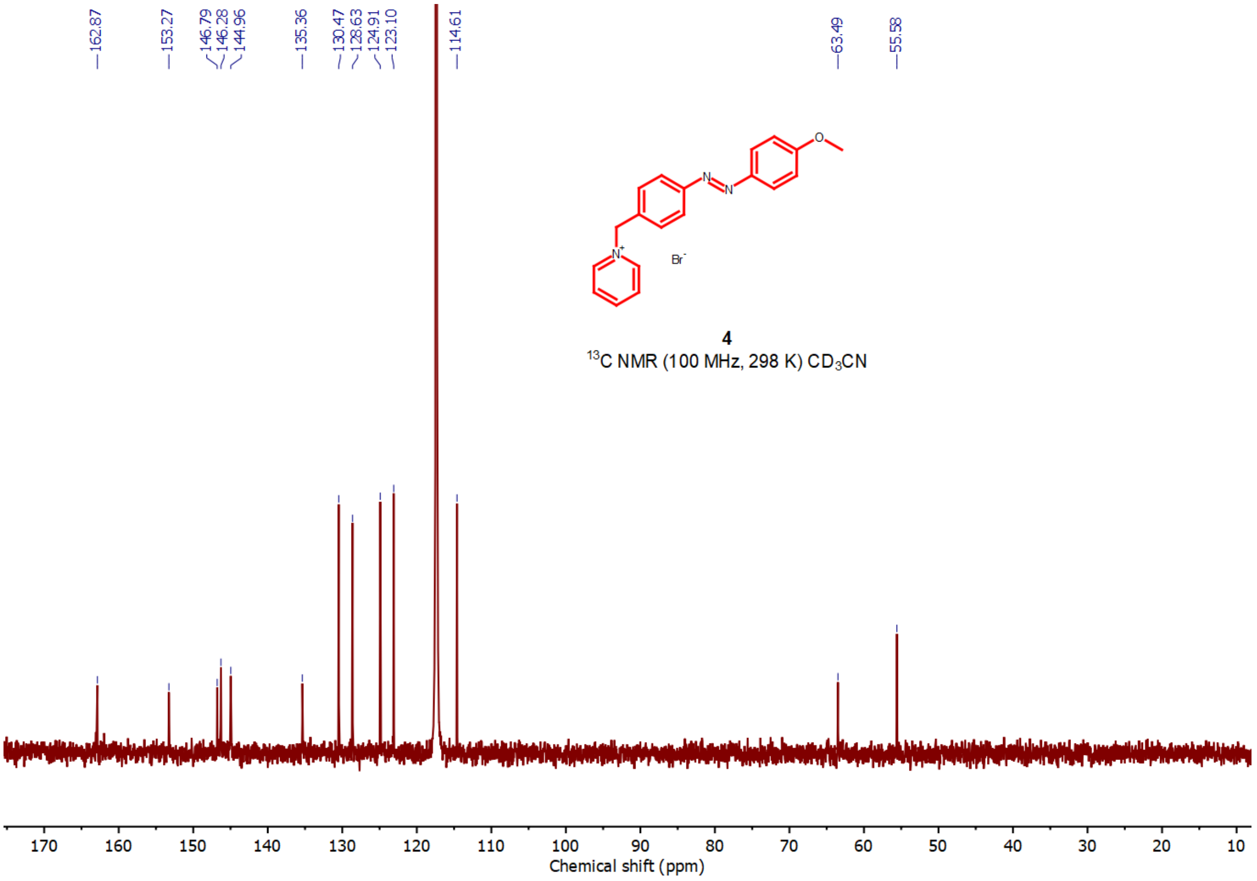

Figure S12: ^{13}C NMR spectrum of **4** in CD_3CN.

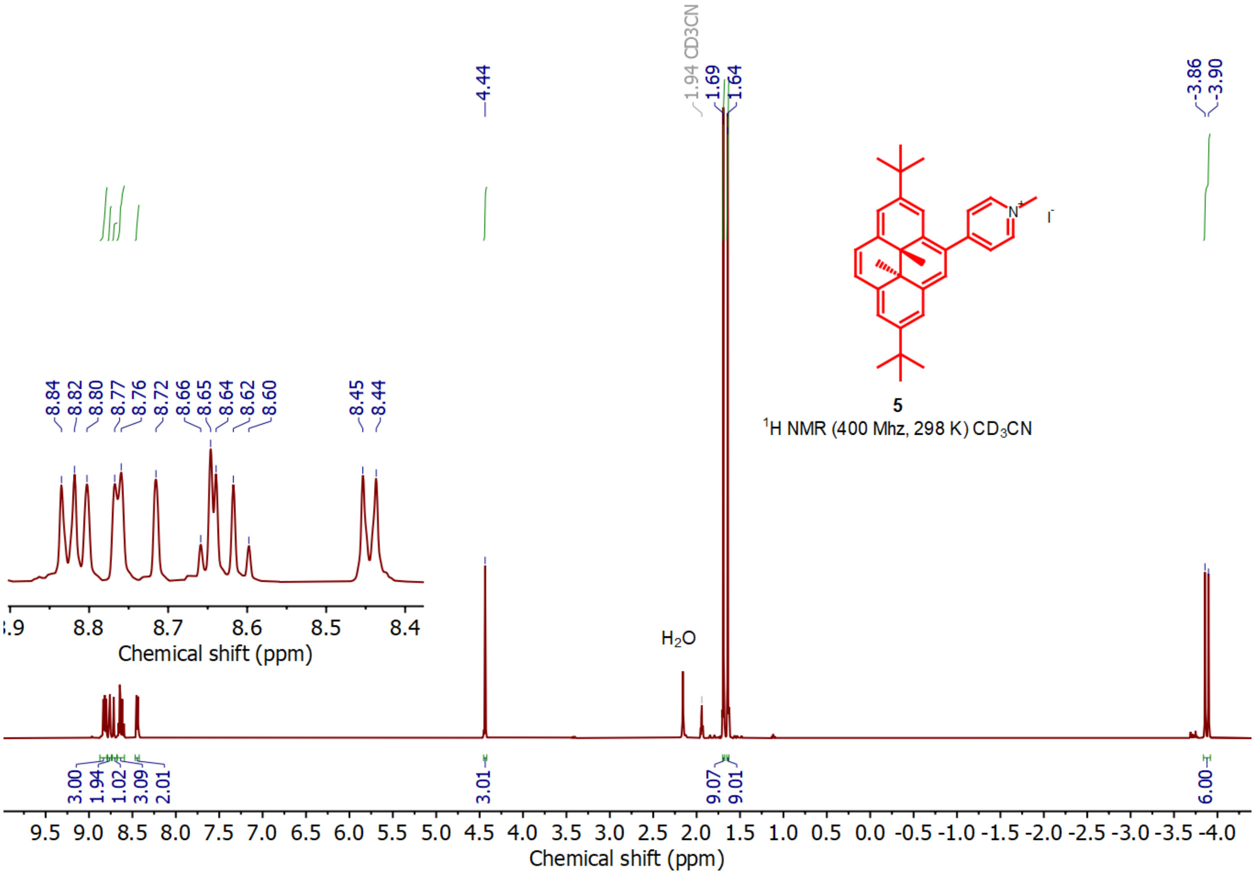

Figure S13: ^{1}H NMR spectrum of **5** in CD_3CN.

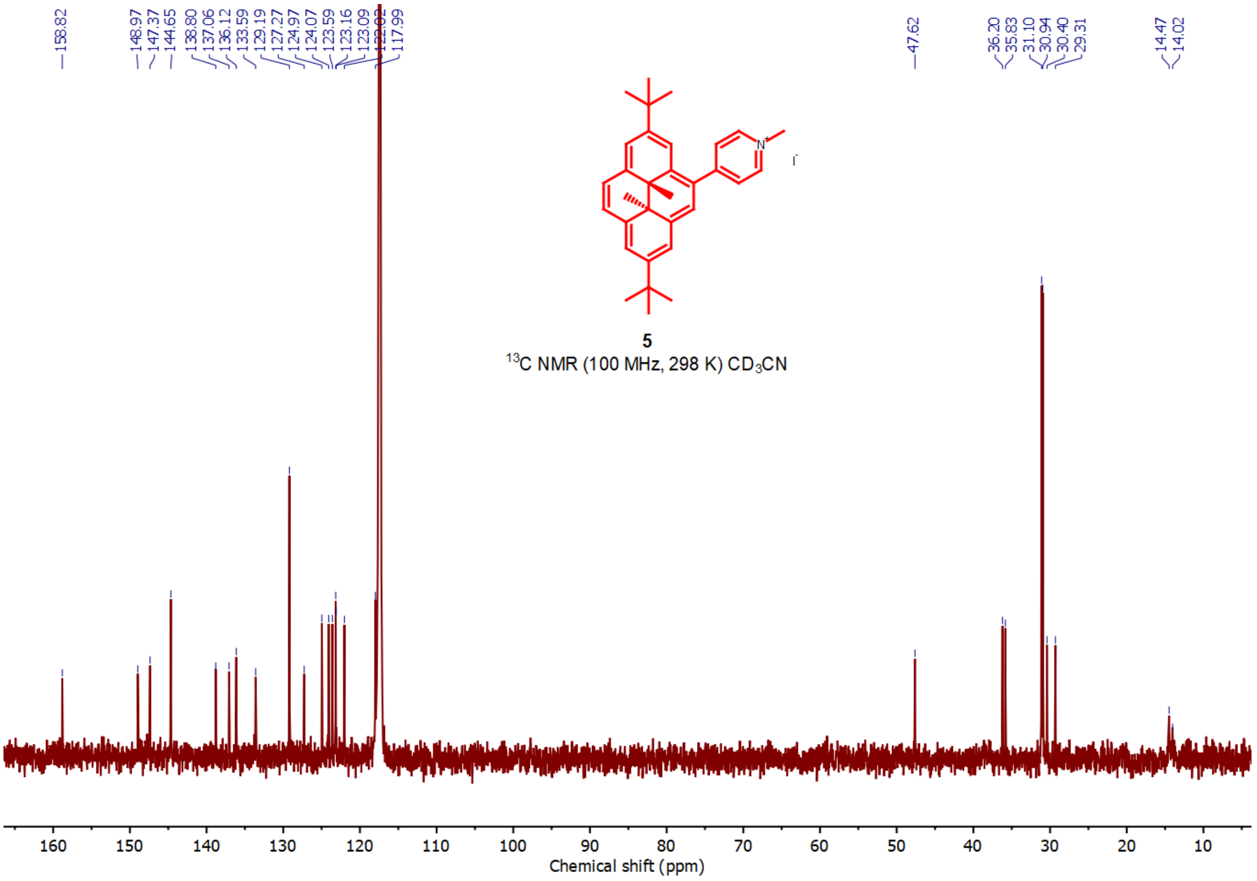

Figure S14: ^{13}C NMR spectrum of **5** in CD_3CN.

1.5 Mass spectrometry data of the synthesized compounds:

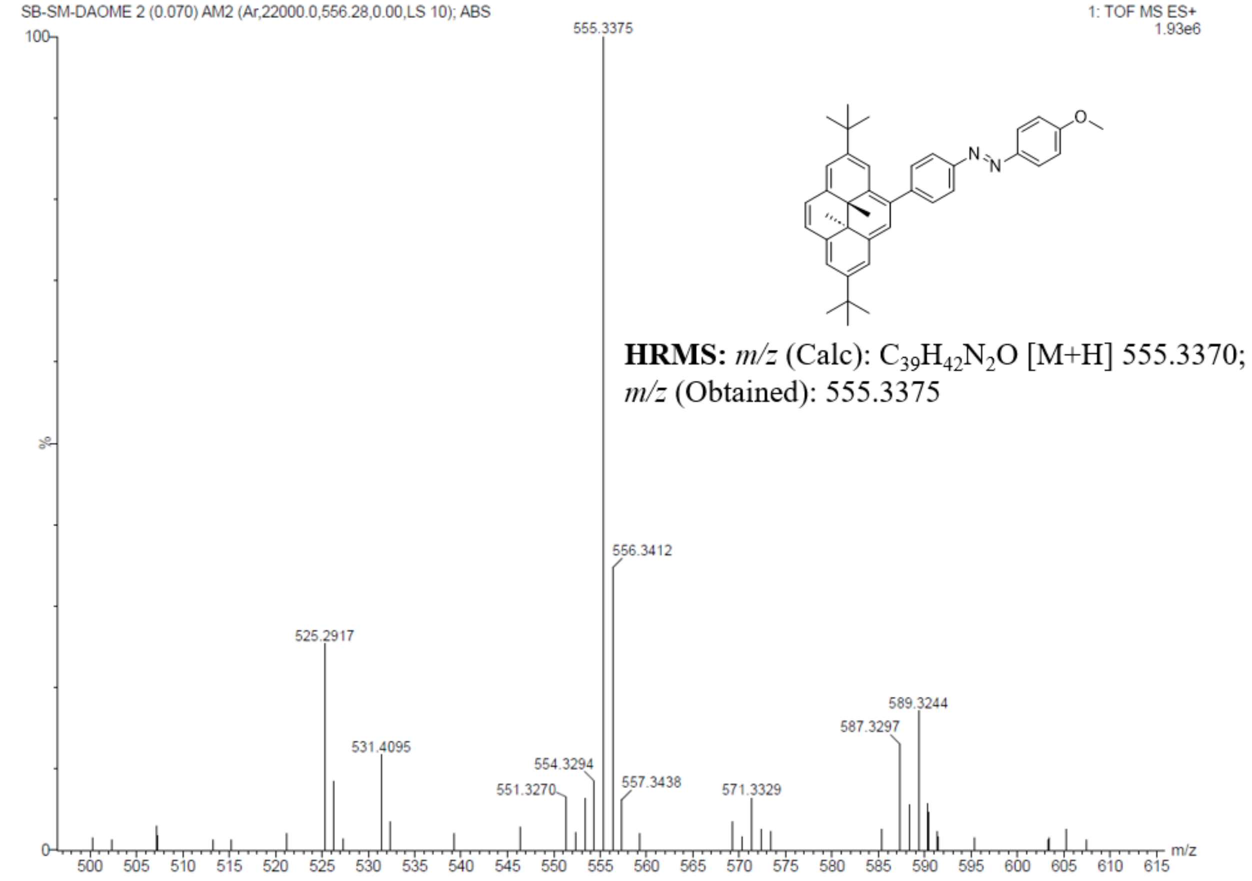

Figure S15: HRMS spectrum of **1**.

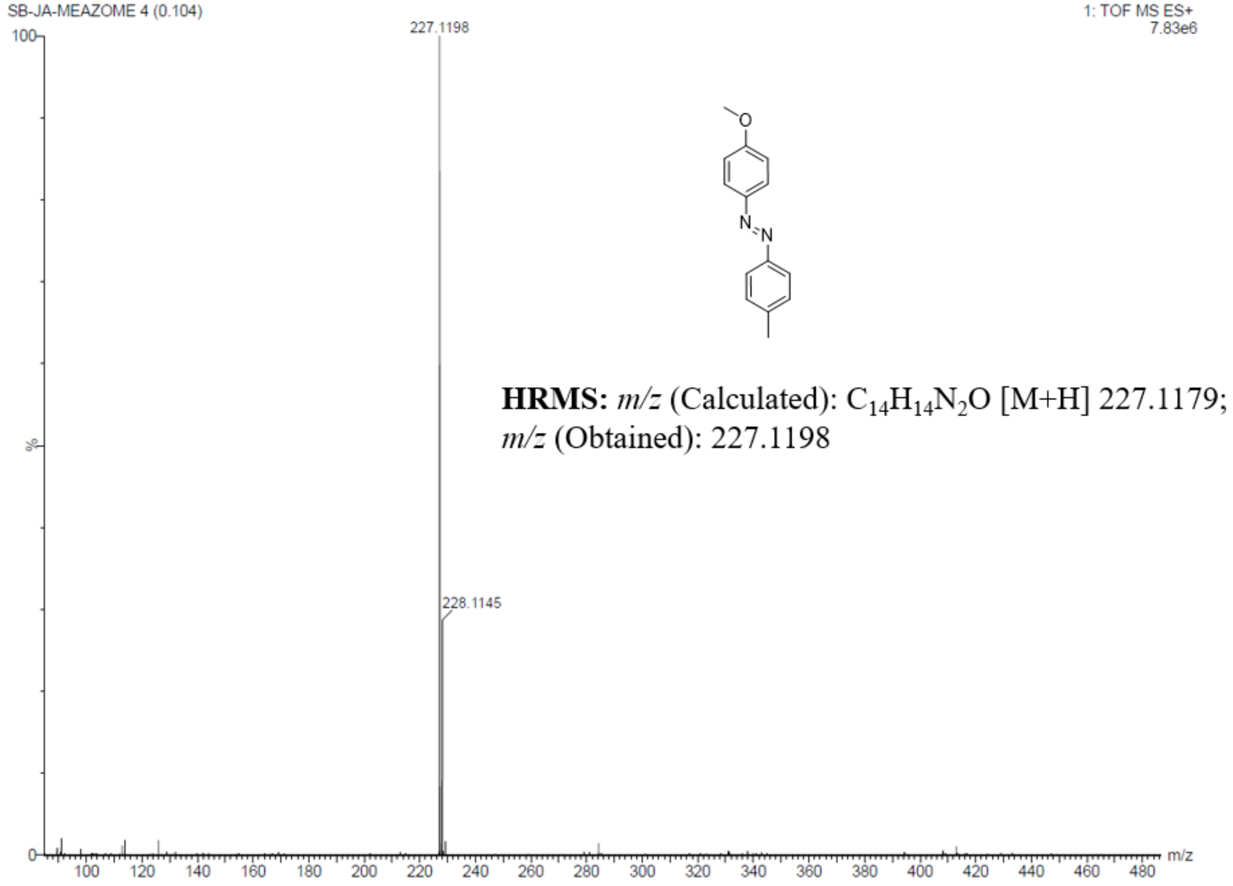

Figure S16: HRMS spectrum of **S2a**.

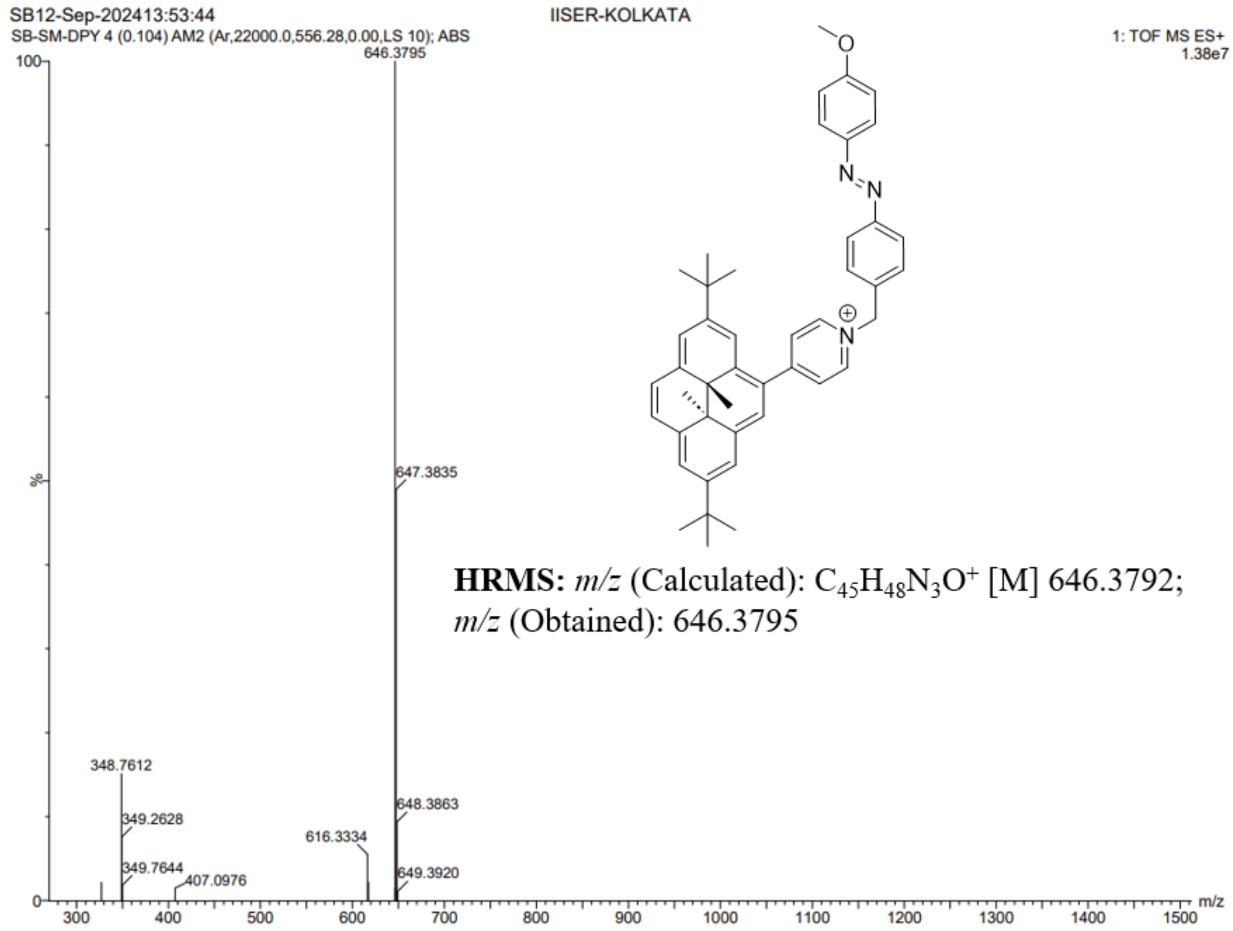

Figure S17: HRMS spectrum of **2**.

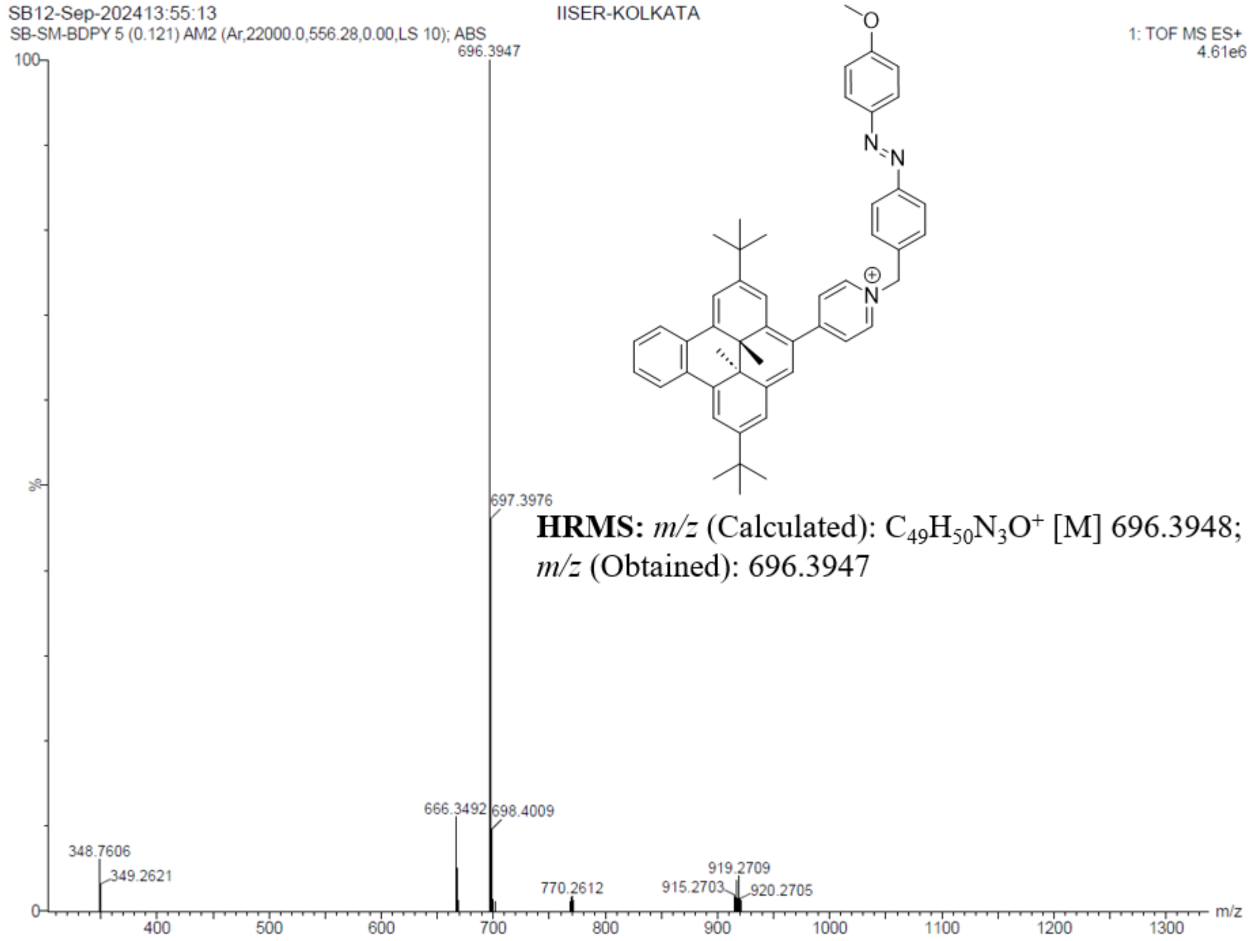

Figure S18: HRMS spectrum of **3**.

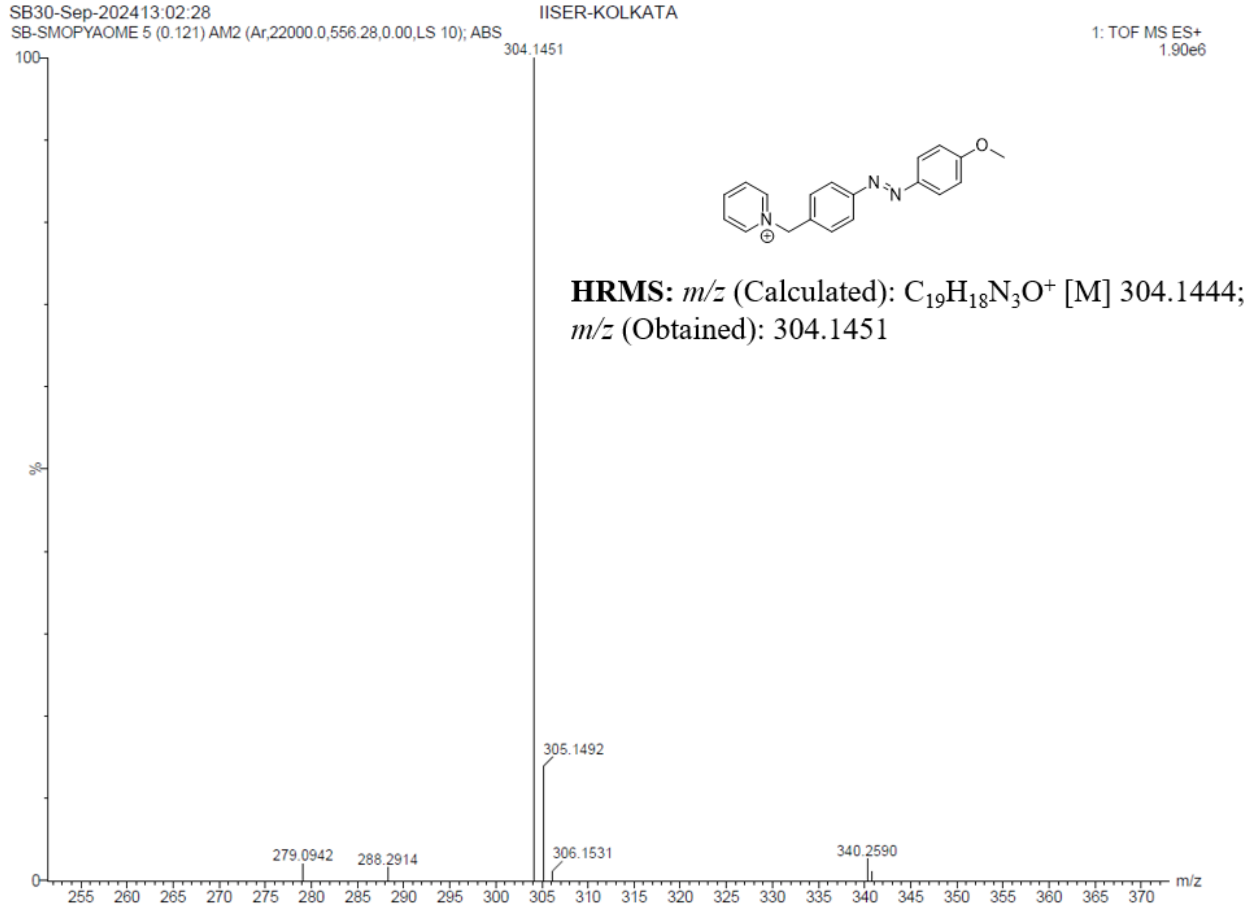

Figure S19: HRMS spectrum of **4**.

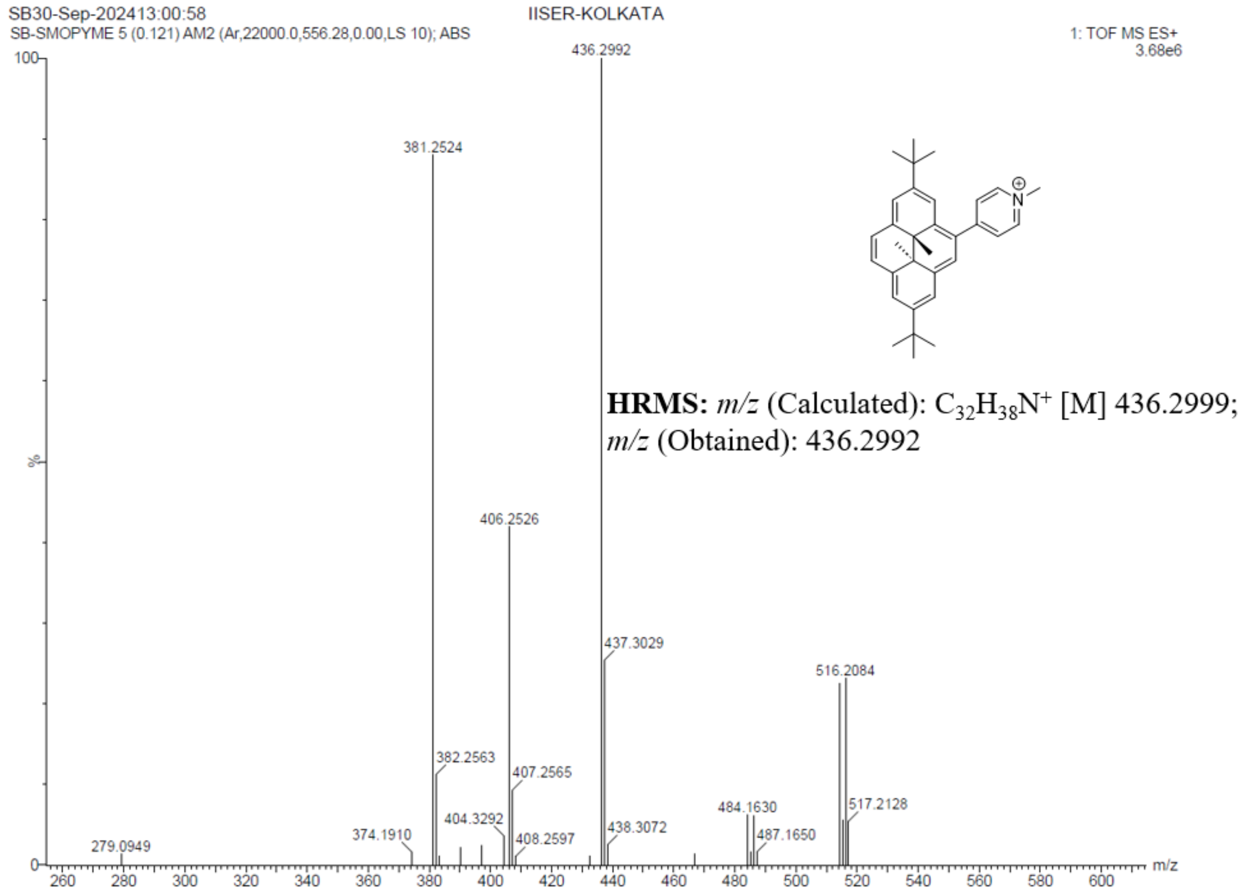

Figure S20: HRMS spectrum of **5**.

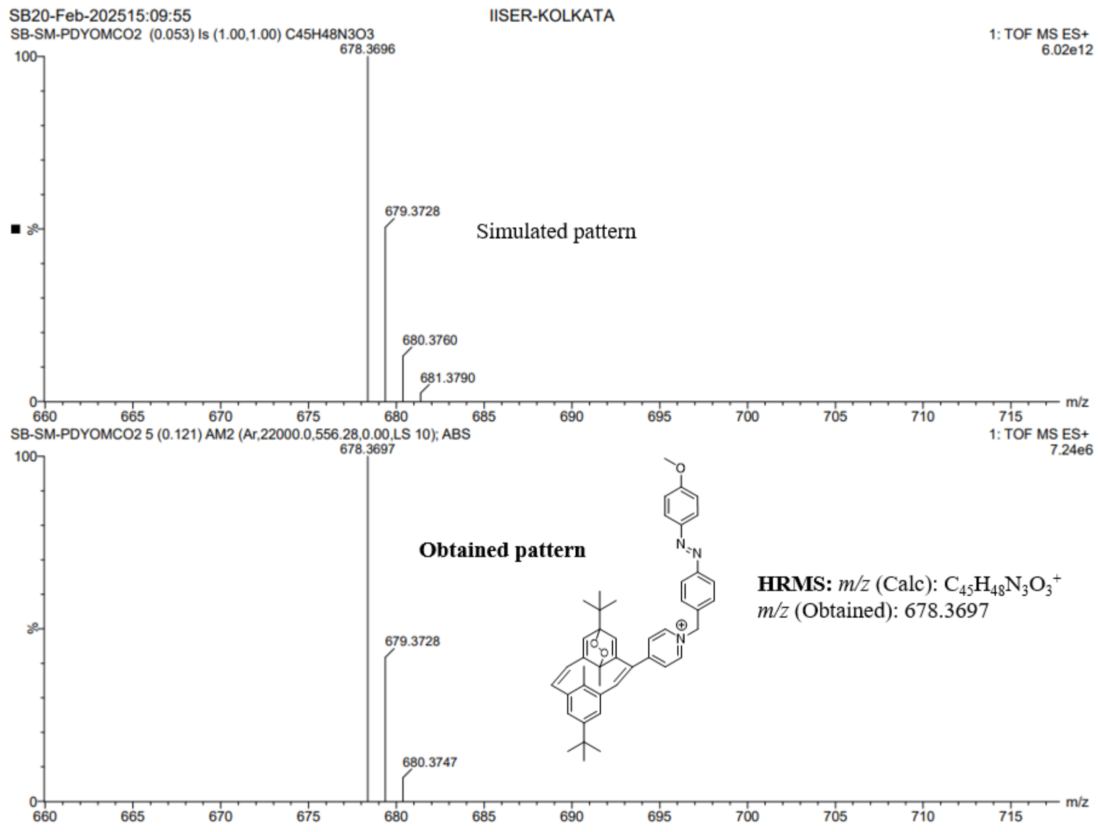

Figure S21: HRMS spectrum of derivative **2** byproduct which has formed upon reaction with atmospheric oxygen upon shining with 640 nm light source.

2. Thermal reversal of compound 1:

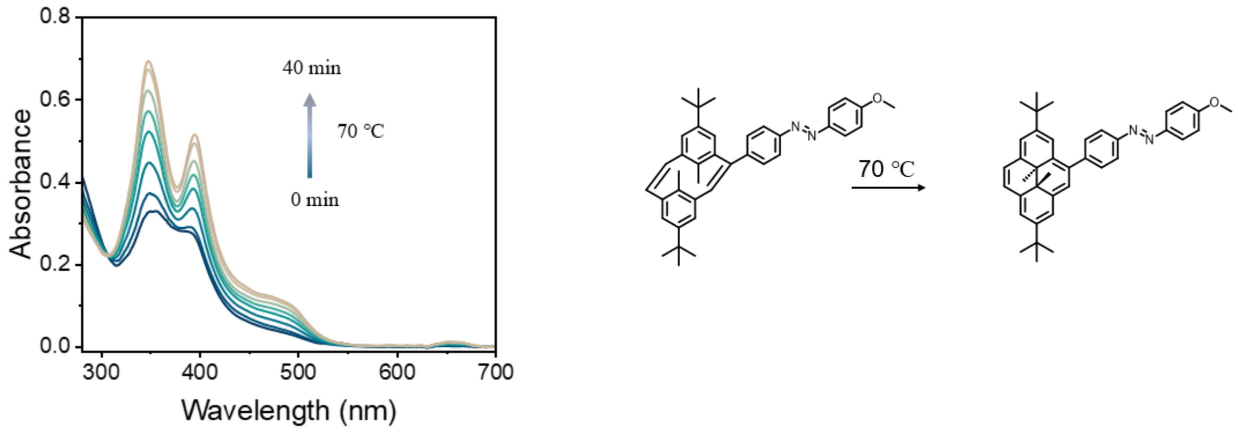

Figure S22: Changes in UV/Vis absorbance upon heating at 70°C for 40 min, showing selective ring closing of the DHP unit under thermal conditions.

3. Control experiments and selectivity:

Control experiments were conducted to evaluate the performance of each individual photoswitch. For **DHPPyMe$^+$**, UV/Vis studies were performed using a 10 µM solution in CH_3CN, while **AzoPyOMe$^+$** was studied in a 20 µM CH_3CN solution (Fig. 4d). The photoisomerization processes were further corroborated through ^1H NMR spectroscopy using 50 mM solutions in CD_3CN. The composition of the photostationary state (PSS) was calculated from the isomerization data obtained using ^1H NMR. For **DHPPyMe$^+$**, the reversal upon irradiation with 456 nm light was confirmed through NMR spectroscopy, validating the successful ring-closing process.

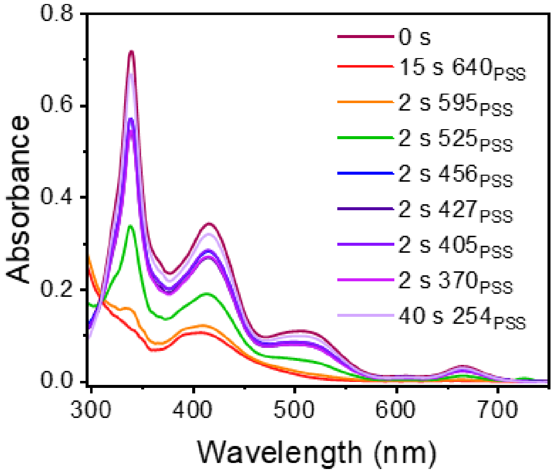

Figure S23: Changes in UV/Vis absorbance of **DHPPyMe$^+$** upon exposure to different lights, showing different PSS of ring closing.

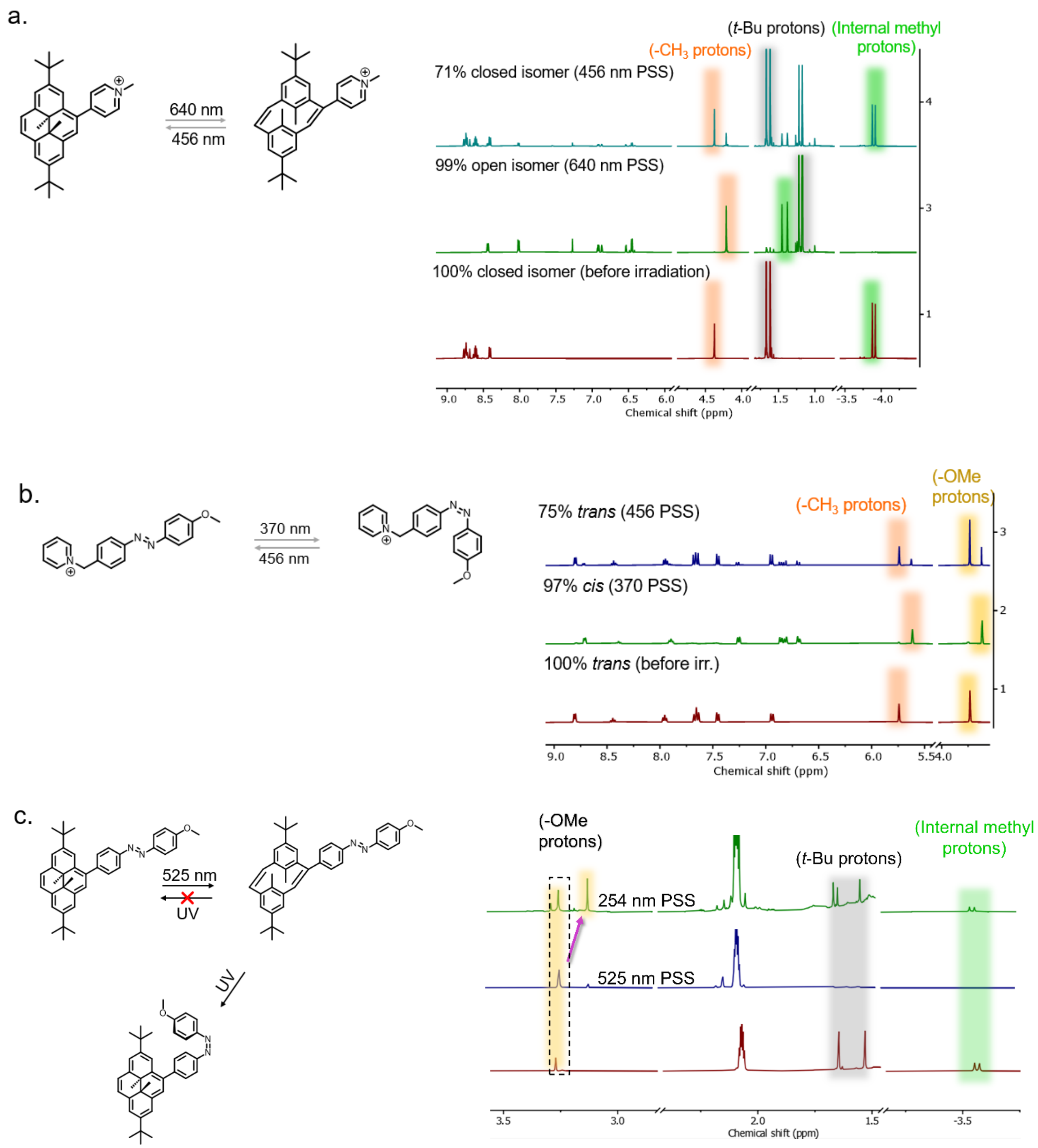

Figure S24: ^{1}H NMR (400 MHz, CD_3CN, 298 K) isomerization spectra of the control compounds: a) **DHPPyMe^{+}** b) **AzoPyOMe^{+}**. 50 mM solutions in CD_3CN were used for the isomerization studies monitored using NMR methods. c) For compound **1**, ^{1}H NMR (400 MHz, 30 mM in toluene-d_8, 298 K) spectra upon photoisomerization was recorded. The reappearance of the signal at –3.5 ppm for the internal methyl protons along with the *t*-Bu protons in the 1.6 ppm region confirmed the ring closing of the DHP unit; the shift of the -OMe protons from 3.23 to 3.12 ppm confirmed the *E*→*Z* isomerization process of the azobenzene unit.

4. Studies of fatigue resistance:

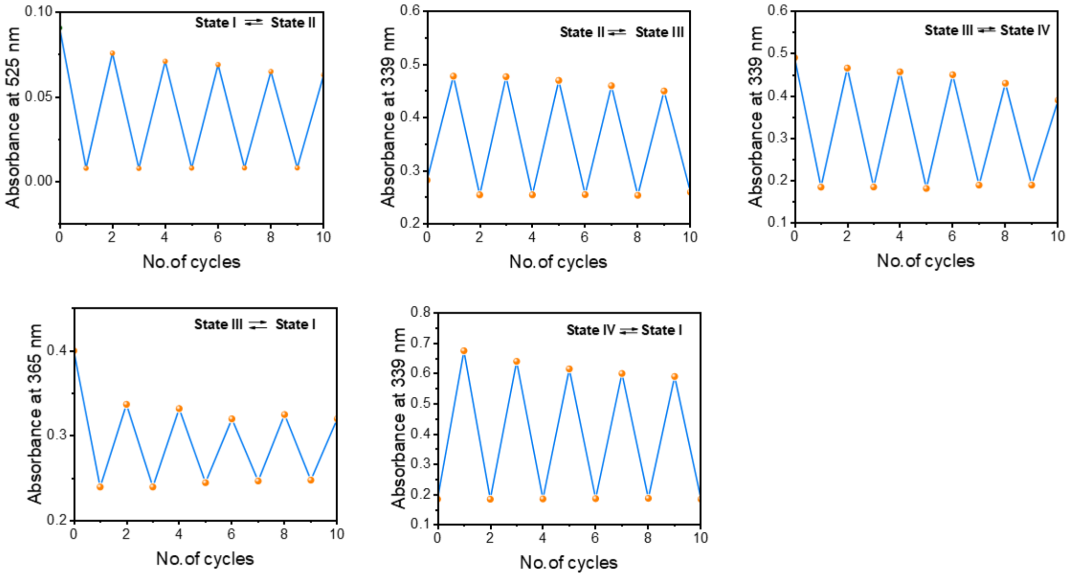

Figure S25: Up to 10 switching cycles for derivative **2** have been monitored upon alternate irradiation with the appropriate wavelength of light (10 μM in CH_3CN). (Observation: the switching step that involves DHP↔CPD isomerization shows a gradual decay in photostability.)

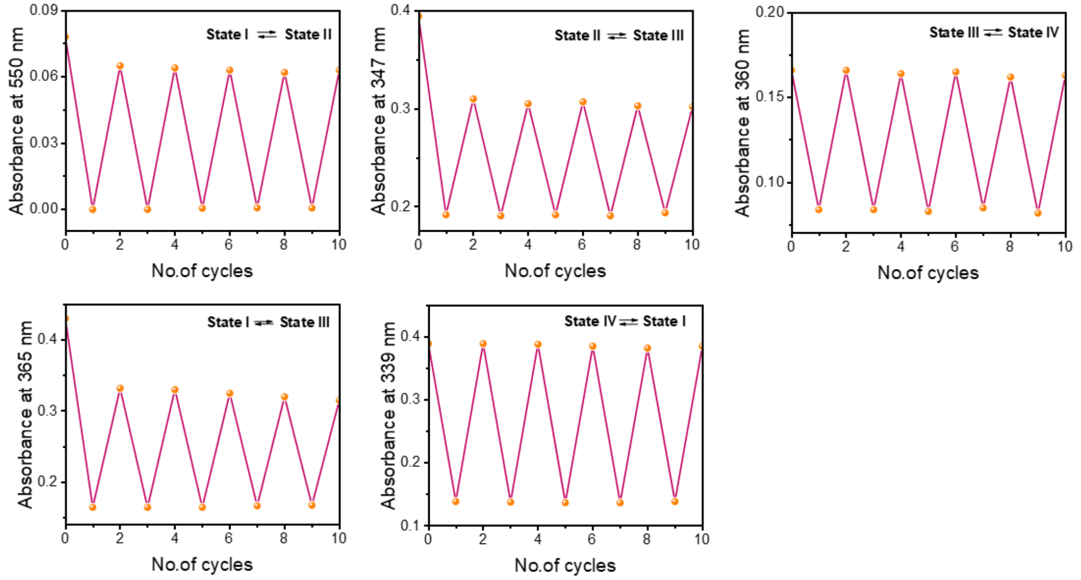

Figure S26: Up to 10 switching cycles for derivative **3** have been monitored upon alternate irradiation with the appropriate wavelength of light. And derivative **3** showcases improved photostability compared to derivative **2** (10 μM in CH_3CN).

5. Photoisomerization quantum yield:

Quantum yield has been calculated using a previously reported method.[6]

The rate of a unidirectional photochemical reaction initiated with monochromatic light is given by:

$$r_{A\to B} = \frac{q_{in}\Phi_{A\to B}}{V}\left(1 - 10^{-\varepsilon_A[A]l}\right) \qquad (1)$$

In our case, since the value of absorbance of the compounds **1** (at 525 nm), and **2** (at 640 nm, wavelength at which the quantum yield was measured) was much less than 0.4, Taylor series expansion of the exponential function and subsequent truncation at the linear term was carried out leading to a first-order rate equation (2). This equation can be further expressed in terms of the quantum yield and the observed first-order rate constant, photon flux and other known measurable quantities for the sample (see below) leading to equation (3):

$$r_{A\to B} = q_{in}\Phi_{A\to B}\varepsilon_A l\, V\, [A] \qquad (2)$$

$$\Phi_{A\to B} = \frac{k\, V}{q_{in}\varepsilon_A l\, ln\, 10} \qquad (3)$$

Where Φ = quantum yield; k= rate constant (obtained from the exponential fit of a graph of A vs. time); V = sample volume; ε_A= molar extinction coefficient; l = pathlength; and q = molar photon flux.

Molar photon flux:

$$q_{in} = \frac{P\lambda}{hcN_A} \qquad (4)$$

where P = power (of the laser); λ = pump wavelength; h = Planck's constant; c = speed of light; and N_A = Avogadro's number.

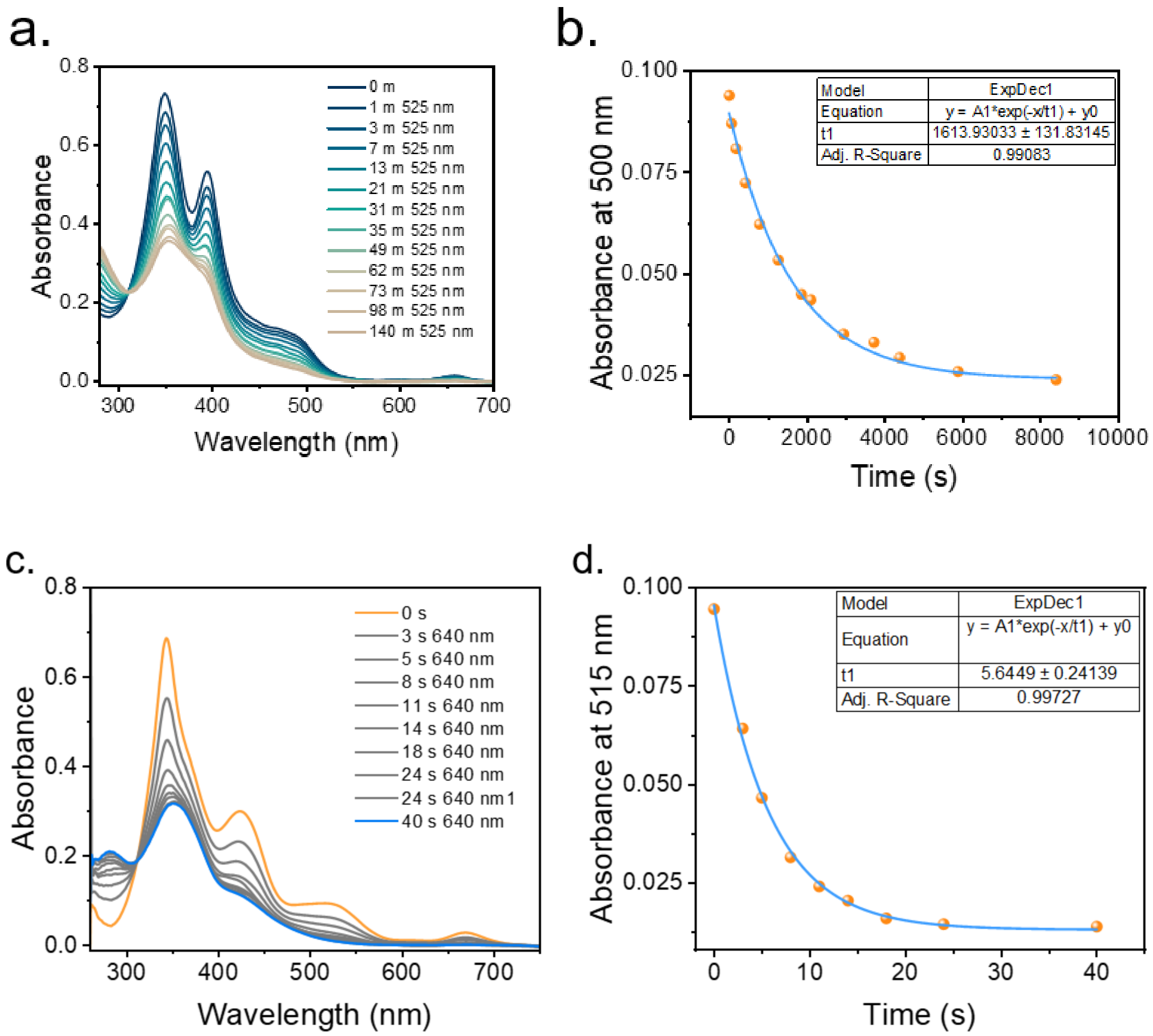

Figure S27: a) Changes in absorbance upon irradiation with 525 nm and b) corresponding kinetics of ring opening photoisomerization of DHP unit for derivative **1**. c) Changes in absorbance upon irradiation with 640 nm and d) corresponding kinetics of ring opening photoisomerization of DHP unit for derivative **2**.

Table S1. Estimation of quantum yields for the forward photoisomerization of in acetonitrile. [5]

Compound	Wavelength (λ/nm)	Extinction coefficient (ε_A) at λ (L $mol^{-1}cm^{-1}$)	Rate constant (k)* s^{-1}	Photon Flux (q_{in}) (10^{-7})	Quantum yield (Φ)
**1**	525	2850±60	0.000619	6.01	1.6×10^{-4}
**2**	640	1300±80	0.177	1.87	0.32

* monitored at low absorbance values of the samples

5. Thermal reversal and kinetic studies:

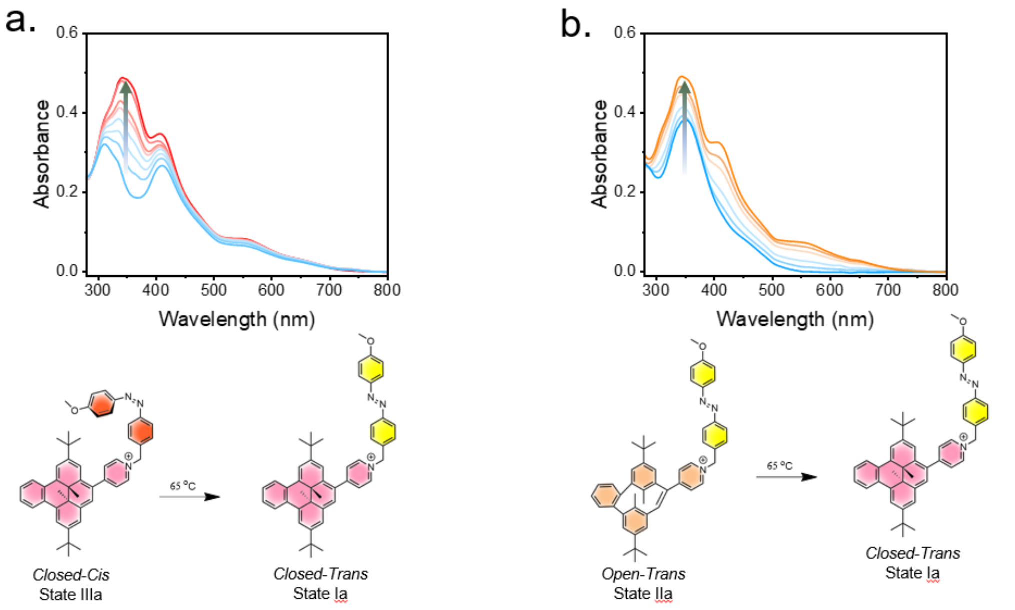

Figure S28: Changes in UV-visible absorbance for the thermal reversal of **3** at 65°C with **(a)** a gradual increment of the band around 367 nm suggesting the $Z{\rightarrow}E$ isomerization of the azobenzene unit and **(b)** the increment of band at the longer wavelength region at around 553 nm suggests ring-closing isomerization.

5.1 Arrhenius and Eyring plots:

The freshly prepared solutions of the photochromic compounds were subjected to exposure of light at distinct wavelengths (525 nm or 640 nm) corresponding to their photochemical characteristics

until a photostationary state (PSS) rich in the photoisomerized open form was attained. To initiate the reverse reaction, the same samples rich in the opened form of PSS were transferred to a dark setting within a Peltier module (temperature specified in the graphs). Absorbance vs time data at each of the temperatures were fitted to exponential plots. The fitted parameters were used to construct the Arrhenius plot that allowed determination of the thermal half-lives of the open forms at 298 K by extrapolation. the rate constants (k) for the thermal reversal were determined using the following equation: $[a] = [a]_0 e^{-kT}$; The half-life, $t_{1/2} = 0.693/k$.

From Arrhenius equation, $\ln k = \ln A - E_a/RT$, a plot of *ln k vs 1/T* yielded the $slope = -E_a/R$. Similarly, from Eyring equation, $\ln (k/T) = -\Delta H^{\neq}/RT + \ln (k_b/h) + \Delta S^{\neq}/R$, a plot of *ln (k/T) vs 1/T* yielded the $slope = -\Delta H^{\neq}/R$ and the $intercept = \Delta S^{\neq}/R$.

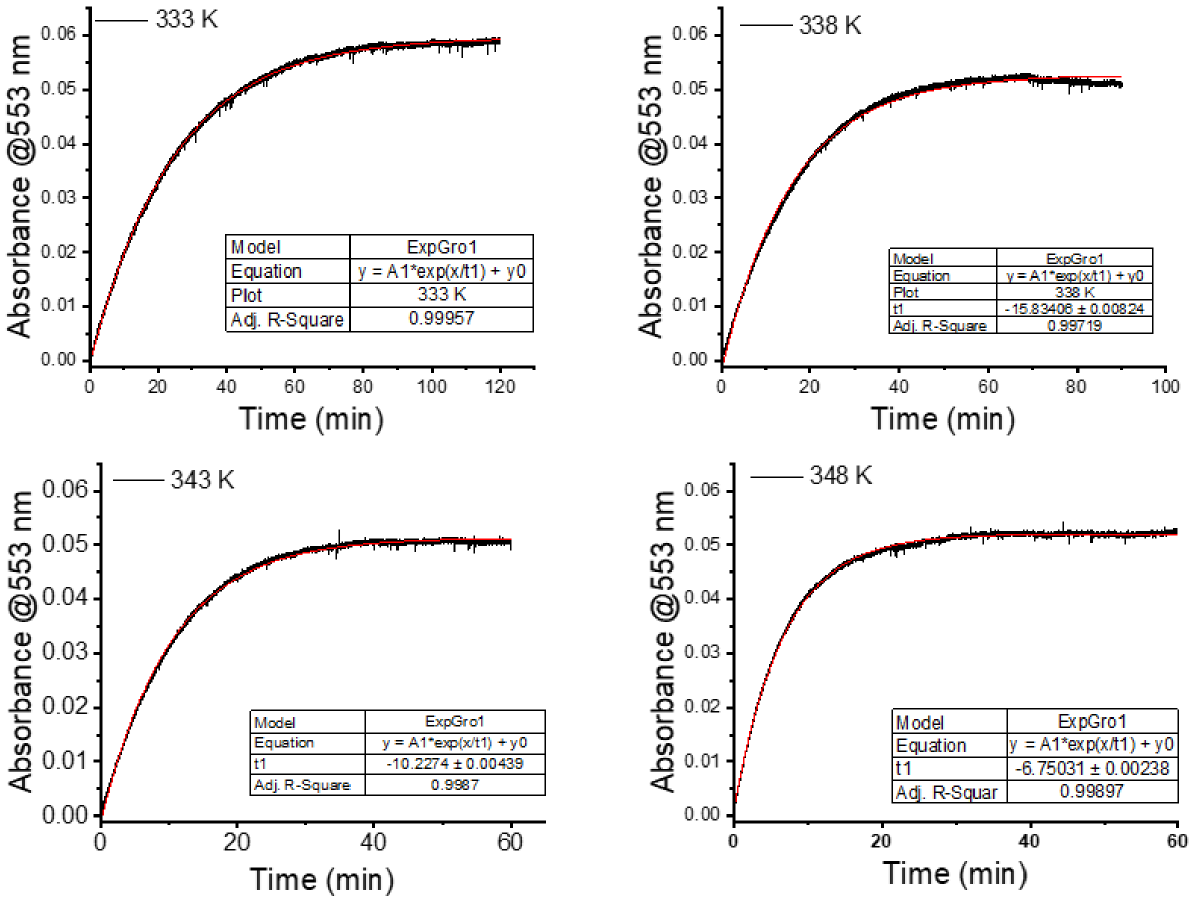

Figure S29: Kinetics plot of compound **3** at variable temperatures (333 K, 338 K, 343 K, and 348 K, respectively) for thermal reversal of the open to the closed isomer. The absorbance at 553 nm at each temperature was fitted to an exponential fit.

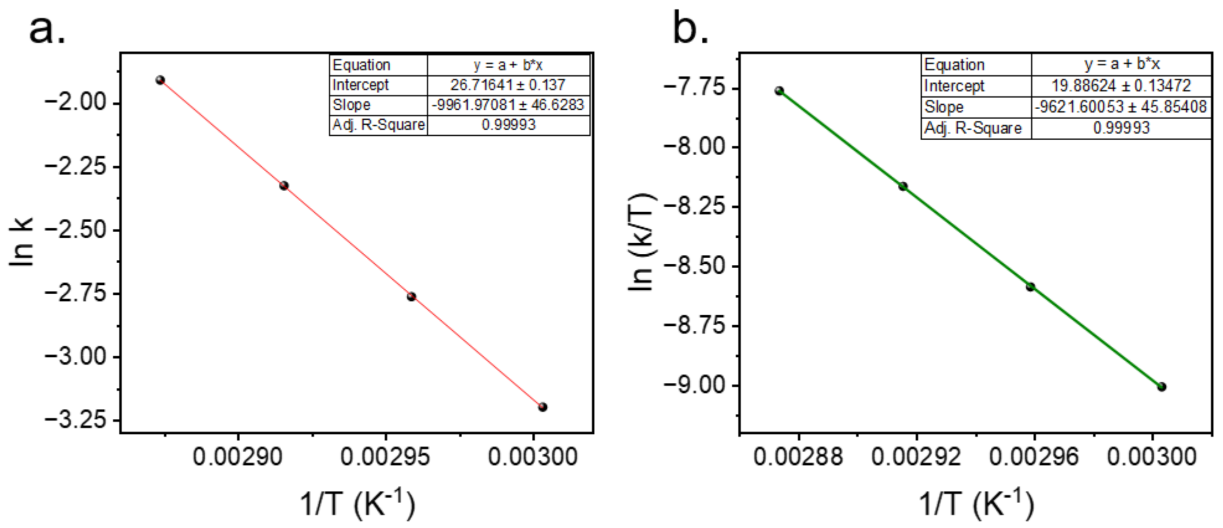

Figure S30: Arrhenius plot and Eyring plot of compound **3** by monitoring absorbance changes at 553 nm.

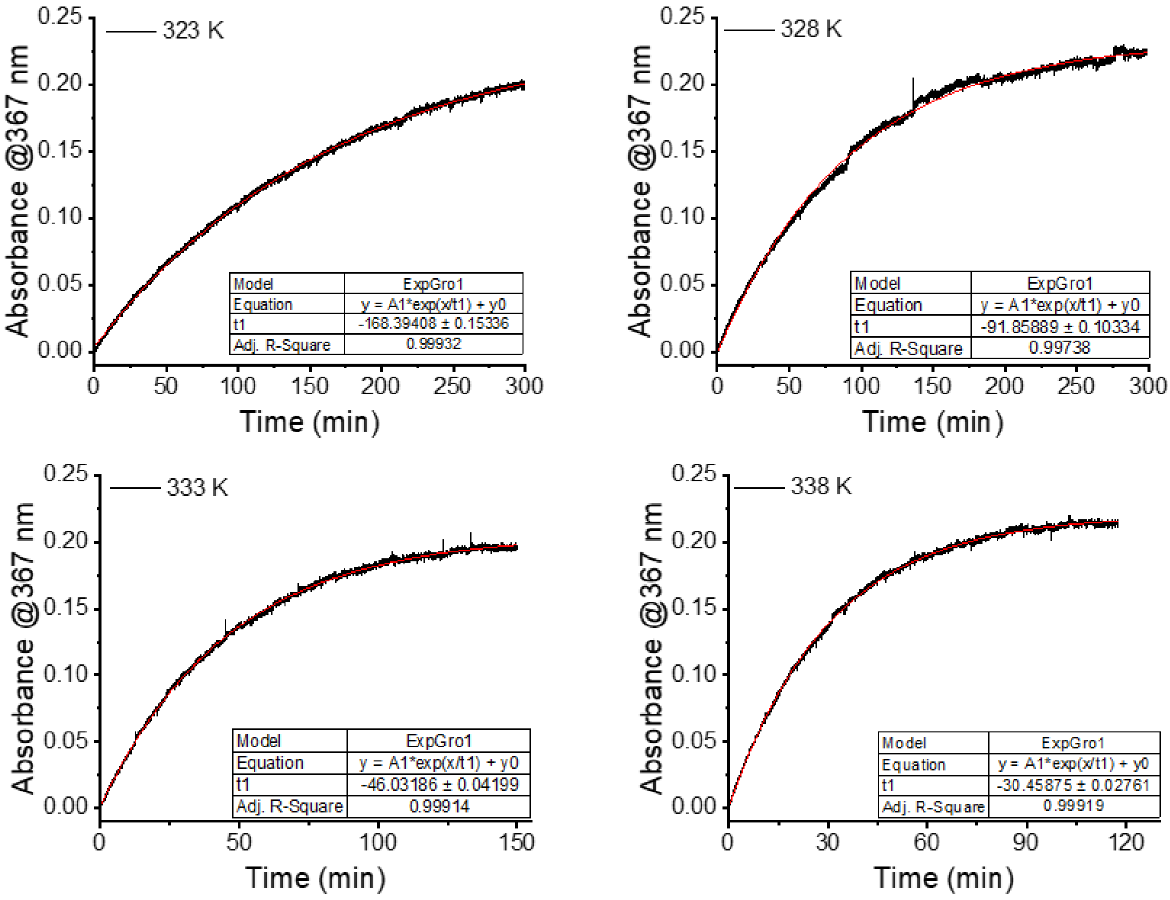

Figure S31: Kinetics plot of 3 at variable temperatures (323 K, 328 K, 333 K, and 338 K, respectively) for the open to closed isomer thermal reversal. The absorbance at 367 nm at each temperature was fitted to an exponential fit.

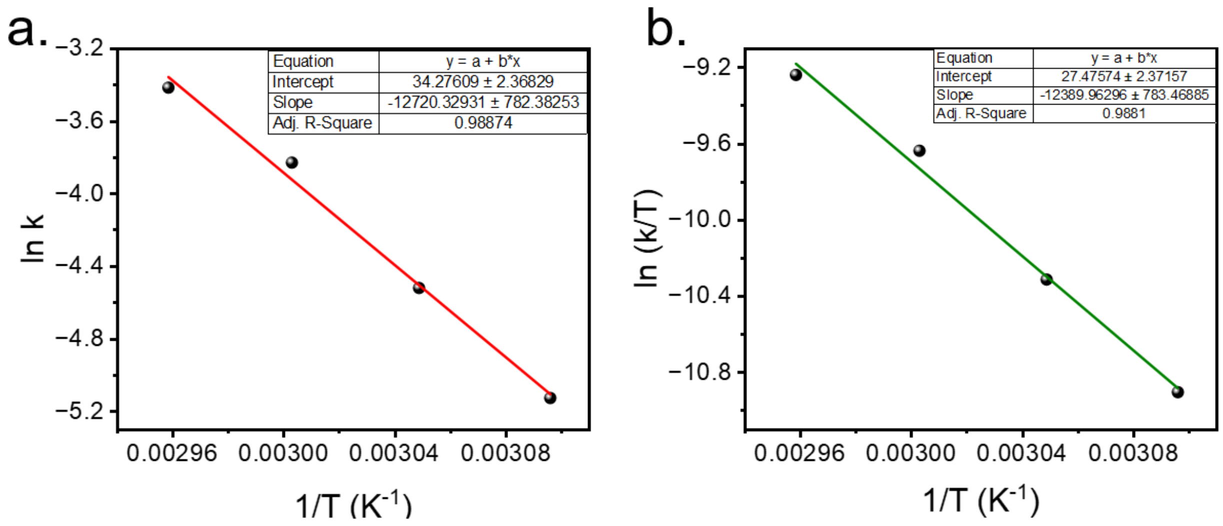

Figure S32: Arrhenius plot and Eyring plot of compound **3** by monitoring absorbance changes at 367 nm.

Table S2. Estimation of thermodynamic parameters for the reverse isomerization of compound **3** for both the ***Z→E*** and ***BCPD→BDHP*** thermal processes in acetonitrile.

**Component**	**Activation energy (E_a) for reverse reaction (kcal/mol)**	**Enthalpy of activation ($\Delta H^{\neq}$) for reverse reaction (kcal/mol)**	**$t_{1/2}$ (h) at 298K**
**BDHP unit** (BCPD→ BDHP)	19.8 ± 0.5	19.1 ± 0.5	9.5
**Azobenzene unit** (*Z*→*E*)	25.3 ± 0.5	24.6 ± 0.5	53

6. Photoswitching studies in thin film:

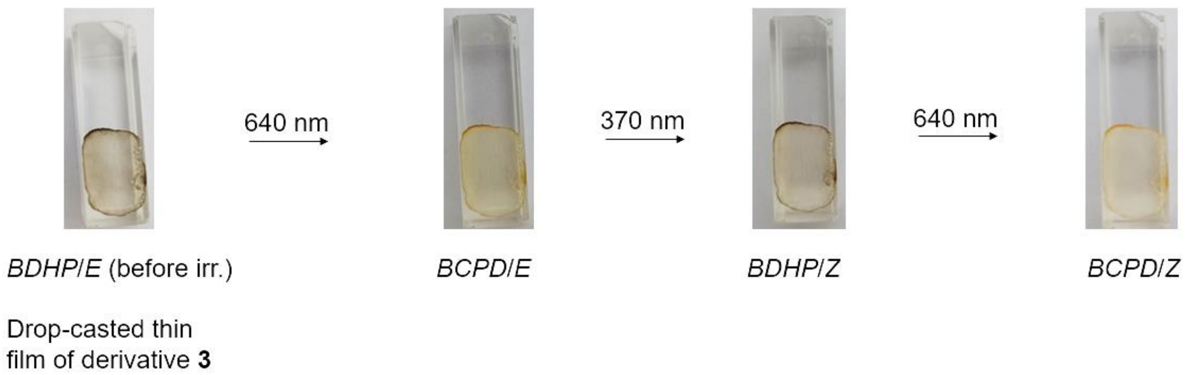

Figure S33: Snapshots of photoisomerization in thin film for derivative **3**.

References:

1. M. Tashiro, T. Yamato, *J. Am. Chem. Soc.,* 1982, **104**, 3701–3707.

2. R. H. Mitchell, Y. H. Lai, R. V. Williams, *J. Org. Chem.,* 1979, **44**, 4733–4735.

3. S. Molla and S. Bandyopadhyay, *J. Mater. Chem. C*, 2024, **12**, 17511–17518.

4. B. Priewisch and K. Rueck-Braun, *J. Org. Chem.,* 2005, **70**, 2350–2352.

5. D. Roldan, S. Cobo, F. Lafolet, N. Vilà, C. Bochot, C. Bucher, E. Saint-Aman, M. Boggio-Pasqua, M. Garavelli and G. Royal, *Chem. - A Eur. J.,* 2015, **21**, 455–467.

6. J. Calbo, C. E. Weston, A. J. P. White, H. S. Rzepa, J. Contreras-García and M. J. Fuchter, *J. Am. Chem. Soc.,* 2017, **139**, 1261–1274.
